# An expanded whole-cell model of *E. coli* links cellular physiology with mechanisms of growth rate control

**DOI:** 10.1038/s41540-022-00242-9

**Published:** 2022-08-19

**Authors:** Travis A. Ahn-Horst, Luis Santiago Mille, Gwanggyu Sun, Jerry H. Morrison, Markus W. Covert

**Affiliations:** Department of Bioengineering, Stanford University, Stanford, CA 94305 USA

**Keywords:** Computational biology and bioinformatics, Microbiology, Molecular biology, Computer modelling

## Abstract

Growth and environmental responses are essential for living organisms to survive and adapt to constantly changing environments. In order to simulate new conditions and capture dynamic responses to environmental shifts in a developing whole-cell model of *E. coli*, we incorporated additional regulation, including dynamics of the global regulator guanosine tetraphosphate (ppGpp), along with dynamics of amino acid biosynthesis and translation. With the model, we show that under perturbed ppGpp conditions, small molecule feedback inhibition pathways, in addition to regulation of expression, play a role in ppGpp regulation of growth. We also found that simulations with dysregulated amino acid synthesis pathways provide average amino acid concentration predictions that are comparable to experimental results but on the single-cell level, concentrations unexpectedly show regular fluctuations. Additionally, during both an upshift and downshift in nutrient availability, the simulated cell responds similarly with a transient increase in the mRNA:rRNA ratio. This additional simulation functionality should support a variety of new applications and expansions of the *E. coli* Whole-Cell Modeling Project.

## Introduction

Since Rudolf Virchow’s declaration in 1855 that “all cells come from cells”, scientists have pursued the question of how child cells arise from a parent: the question of growth. In particular, while it is now widely known that cells take in nutrients from the environment, grow, and give rise to new cells, what is less well understood is the dynamics of how cells can respond to nutrients, regulate gene expression, and optimize growth when faced with changing environments. A cohesive, integrated, and ideally, predictive theory of cell growth is thus one of the oldest and most fundamental pursuits in the field of biology, not only in terms of basic science but also with a host of potential applications.

**Fig. 1:**
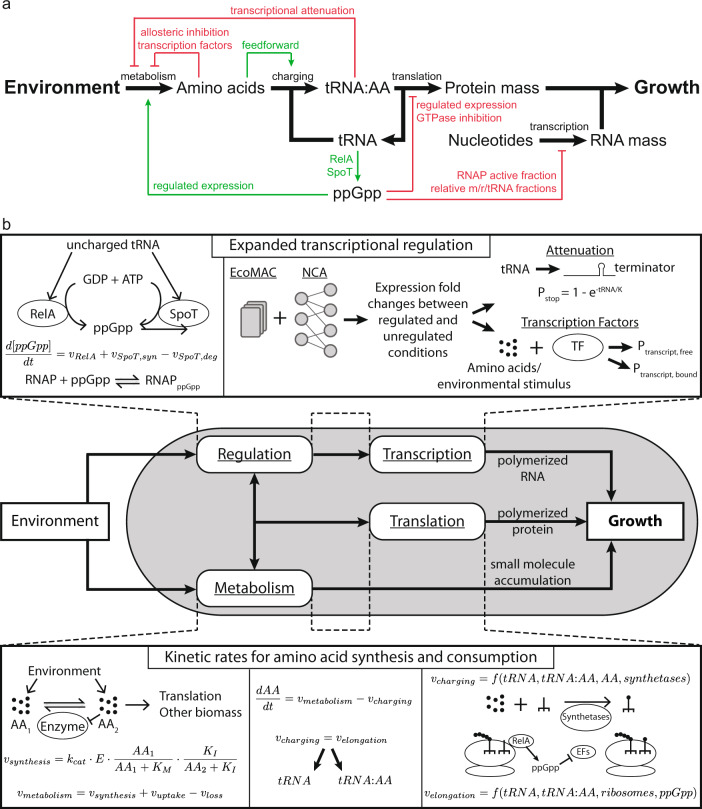
A detailed, holistic model of growth rate control has been incorporated into the *E. coli* Whole-Cell Modeling Project. **a** A schematic representing biological functions and regulation that link the environment to growth. Black arrows represent mass flow, red arrows indicate regulatory inhibition and green arrows represent activation. **b** A schematic illustrating the integration of the biological functions in (**a**) in the context of the *E. coli* whole-cell model by including regulatory interactions and kinetic reaction rates. Such integration allows the growth rate to be determined by the simulation state, which is responsive to the simulated environment of interest. Dashed lines represent the link between the new mathematical representations and existing modeling processes that were modified. The resulting model includes more gene functions, accounts for the action of more small molecules, and can accommodate simulations in more environments.

A large body of work has sought to describe the determinants of growth, often starting with a coarse-grained perspective by capturing relationships that are observed empirically. Often, this starts by relating ribosome content to growth rate and considering the limits translation places on growth^1–3^, which can also be extended to dynamic environments^4,5^. Some have also considered the trade-offs a self-replicating system must make when constrained by resource allocation^6–8^. Still others have used more fine-grained models to probe the mechanisms that underlie growth, such as guanosine tetraphosphate (ppGpp) regulation of ribosome expression^9^. In this work, we sought to represent knowledge learned from these previous studies (Fig. 1a) in the context of a developing whole-cell model, not only to expand the functionality and predictive capabilities of the model, but also to provide a more integrated framework for assessing the molecular underpinnings of growth control and responses to changing environments.

Whole-cell modeling is an approach that facilitates comprehensive mechanistic modeling and is also readily applicable to empirical analysis. By choosing mathematical representations of physiological subunits of the cell (eg. transcription, metabolism, protein degradation) that are most suited to the collective understanding of, data availability for, and dynamics of these individual processes, whole-cell modeling can track and update the internal state of a simulated cell as it grows. This highly integrative method can therefore bridge both mechanistic and empirical approaches, and in the process can provide a deep understanding of changes that happen throughout the entire cell. Whole-cell modeling was first conceived in the 1970s by Francis Crick^10^, followed by Michael Shuler^11^ Harold Morowitz^12^ and Masaru Tomita^13^, but it was first demonstrated decades later in *M. genitalium*, the smallest culturable organism^14,15^, and has more recently been applied to *E. coli*^16,17^. Using whole-cell models to capture behavior of single cells allows for integration and assessment of heterogeneous datasets and the scientific community’s current understanding of biological interactions through mechanistic relationships. Although the original *E. coli* whole-cell model described in Macklin et al.^16^ accounted for many of the processes and physiology of *E. coli*, including the central dogma, metabolism and regulation, the *E. coli* Whole-Cell Modeling Project^17^ is an ongoing effort to expand the scope of the *E. coli* whole-cell model by including missing gene and small molecule functionality as well as increasing the number of possible nutrient conditions for simulated growth.

A top priority of the *E. coli* Whole-Cell Modeling Project is the ability to accurately simulate cell growth in a variety of environmental conditions, and central to this priority is a detailed model of growth rate control. Although the benchmark for the original whole-cell model was that it be “gene-complete”, the *E. coli* model should not only account for the known functions of all the genes but also be able to respond to any environmental condition. Fortunately, *E. coli* has already been characterized in many environmental conditions, which provides a basis for defining and parameterizing a whole-cell model. Prior to this work, the *E. coli* model was parameterized for growth on three separate environments: defined rich media, minimal glucose media and anaerobic minimal glucose media. These environments were essentially incorporated as three distinct versions of the model without detailed control. As a result, simulating transitions between conditions, while possible, was not realistic. Including mechanistic regulation in response to changing environments in a simulation would allow the model both to accommodate more environments and also to simulate cell states in between defined media conditions. Such transition behaviors are far less frequently observed experimentally, and so we hoped that the model might provide mechanistic insight into cell growth in dynamically shifting environments.

In this study, we look to extend the previous version of the *E. coli* whole-cell model by including additional regulation and dynamics in order to more accurately capture growth and environmental responses. ppGpp is a major regulator of growth in *E. coli*^18^ and controls the stringent response to nutrient limitation. ppGpp is produced from GDP and ATP by RelA and SpoT and concentrations rise in response to nutrient limitations. Although ppGpp plays a role in many cellular processes^19^, one of the key areas of influence is by interacting with RNA polymerases (RNAP) to downregulate rRNA, tRNA, ribosomal proteins and tRNA synthetases, while upregulating amino acid synthesis enzymes and stress response genes. Transcription factors play a more targeted regulatory role in responding to specific environmental changes^20^, and can fine tune expression to optimize growth in certain conditions, for example, ArcA and Fnr responding to anaerobic conditions^21^ or Crp and Cra coordinating central carbon metabolism expression^22^. There have been separate attempts to model both ppGpp regulation of growth^9^ and transcription factor effects on growth in various media conditions^23^. The *E. coli* Whole-Cell Modeling Project provides a basis for integrating both together along with more detailed models of the central dogma for dynamic responses to environmental changes.

In *The Model Thinker*, Scott Page uses the acronym REDCAPE to capture all of the possible uses for a model: Reasoning, Explanation, Design, Communication, Action, Prediction, and Exploration^24^. In that context, some of the primary goals of this work were to use a detailed model of growth rate control in *E. coli* to *explain* in more detail the mechanistic underpinnings of the relationships and “laws” that have been identified and affirmed in recent studies, as well as to *explore* how such mechanism comes together in the case of a nutrient shift. We discuss our model, the resulting simulations and the new findings that resulted from them in detail below.

## Results

### An expanded *E. coli* model incorporates major determinants of growth rate

In order to simulate new conditions and capture dynamic responses to environmental shifts, our new version of the model includes additional biological functionality and incorporates more experimental data. Translating the biological function into mathematical representations used in the model required the addition of two modeling modules: (i) expanded transcriptional regulation and (ii) kinetic rates for amino acid synthesis and consumption (top and bottom panels of Fig. 1b). These new features are integrated into the existing model framework by updating physiological process submodels of the *E. coli* whole-cell model to sense environmental changes, alter the internal state, and ultimately give rise to mechanistically determined growth rate in the simulation (center panel of Fig. 1b). While the previous version of the whole-cell model included fixed rates of RNA and protein polymerization for specific environmental conditions based on literature values^16^, the new submodels provide a mechanistic approach to growth where these rates are determined by the internal state of the simulation (see “Methods” for complete details of new submodels, as well as how all simulations were run and analyzed to create the figures).

With regard to expanding the transcriptional regulation component of the model, we began by adding a kinetic submodel of ppGpp synthesis and degradation, a central regulatory molecule in transcription-based growth rate control. The concentration of ppGpp over time is represented as an ordinary differential equation accounting for kinetic reaction rates of the RelA and SpoT enzymes, which depend on enzyme expression levels, the amount of uncharged tRNA and the ppGpp concentration itself with kinetic parameters based on measured values reported in the literature (see “Methods”). With a dynamic ppGpp concentration, the model can then capture the numerous transcriptional regulatory effects ppGpp exerts through binding with RNA polymerase. The destabilizing effect that ppGpp has on RNAP and the open complex during transcript initiation leads to changes in the fraction of RNAPs that are actively transcribing as well as changes to gene expression (including downregulation of ribosomal genes and upregulation of metabolic enzymes), which in turn alters the fractions of mRNA, rRNA and tRNA that are produced. Changes to the fractions of mRNA, rRNA and tRNA being transcribed can also lead to changes to the average RNAP elongation rate due to differences in the elongation rates between mRNA and stable RNA. By incorporating the regulation of RNAP properties by ppGpp, we hoped to capture more dynamics of RNA polymerization based on the environment and internal state in our simulations, which also contributes to the overall dynamics of the cellular growth rate.

Beyond ppGpp regulation of gene expression, we introduced additional transcriptional regulation to the model, namely, greatly expanding the control of transcription factors and including transcriptional attenuation, as summarized in Supplementary Table 1. Parameterization of the new regulation was achieved using network component analysis (NCA)^25–27^. NCA is a matrix decomposition method that can provide regulatory interaction strengths between individual regulators and genes along with overall activity signals in a measured condition based on a set of expression data measurements and subject to constraints of the known interaction network topology. Inputs to NCA were transcriptomics data from EcoMAC^28^ and known regulatory interactions from EcoCyc^29^. The output provided fold changes that were consistent with both the transcriptomics and the known regulatory network and were used to parameterize additional transcription factor-to-gene regulatory interactions in the model for 785 (17% of all genes) new genes that were previously unregulated in the whole-cell model. Using NCA also allowed for the addition of a model of transcriptional attenuation by providing a way to parameterize the probability of attenuation through the calculated fold changes. In total, the newly added regulation provides the differential expression of 1052 (23% of all genes) additional genes, consistent with annotated regulation, in order to more accurately simulate new environments.

With regard to the second module, detailed rate equations for the production and consumption of amino acids (based on Michaelis-Menten kinetics) were also added to the model. The synthesis rates are controlled by the expression of synthesis enzymes, the concentration of upstream amino acids and the concentration of the amino acid end product, which provides feedback either through allosteric inhibition or a reversible reaction. The model of amino acid transport was also expanded to include mechanistic import and export rates based on internal amino acid concentrations and transporter expression. Additionally, loss rates to other metabolic reaction pathways are defined with kinetic rates. Taken together, this means that the net rate of amino acid production from metabolism can be determined based on the internal state of the simulated cell and the environment. The rate of supply of amino acids to elongating peptides involves the charging of tRNA and polypeptide elongation at the ribosome, and kinetic rates for both of these processes were also added to the model. This addition more directly ties the output of translation, one of the major drivers of growth rate, to the internal simulation state, and combined with the amino acid synthesis rates, produces dynamic amino acid concentrations in the simulation.

### Simulation outputs are a more realistic representation of *E. coli* physiology

**Fig. 2:**
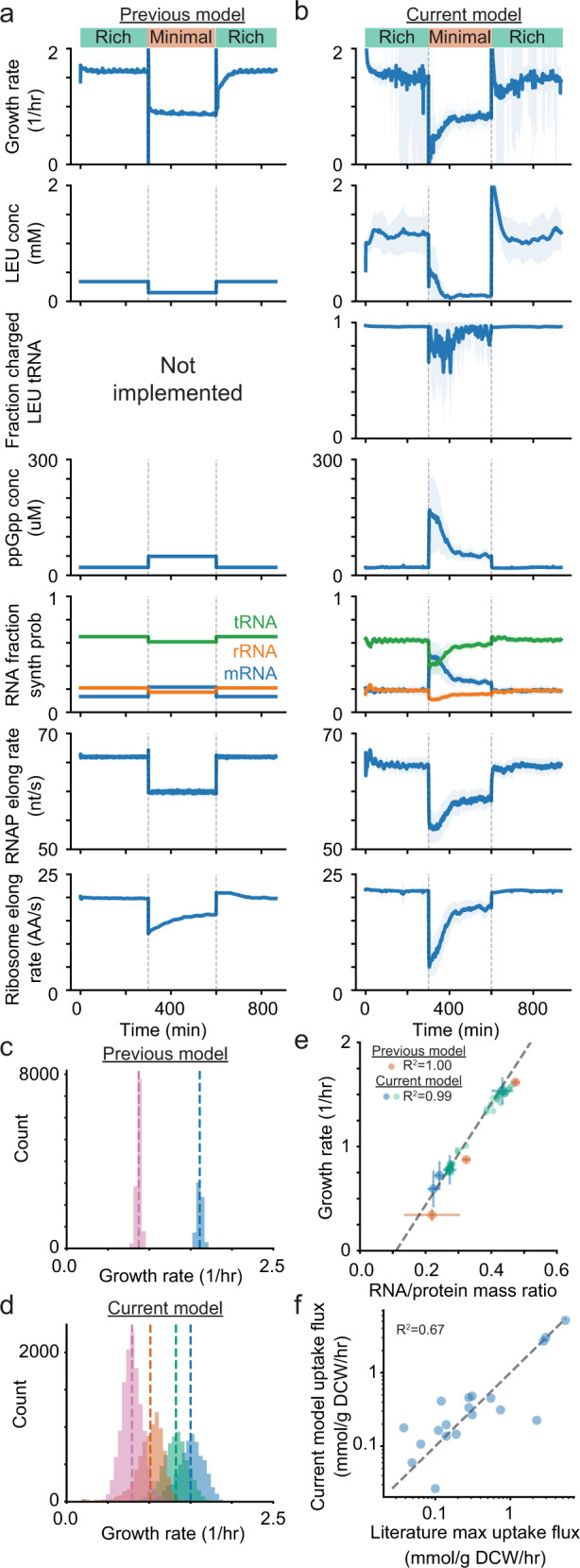
Incorporation of the growth rate model results in simulation responses to environments and environmental shifts that are more accurate for well-characterized environments, and more biologically reasonable in less well-characterized environments. Time series data show a nutrient downshift (green to orange) following by a nutrient upshift (orange to green) over 28 cell generations in the previous model (**a**) and the current model (**b**). The mean of 32 initial seeds is shown as the dark blue line with the light blue region showing the standard deviation. **c** Distributions of growth rates from 24 generations and 24 initial seeds from the previous version of the model in minimal (pink) and rich (blue) media with dashed lines showing the mean value. **d** Distributions of growth rates from 24 generations and 24 initial seeds from the current version of the model with new conditions (orange and green) that are not directly parameterized and include arbitrary amino acid combinations in the media. **e** Relationship between growth rate and RNA/protein ratio for multiple environmental conditions. The three possible media conditions from the previous model are shown in orange, new media conditions used for parameterization (and simulated) are in blue, new media conditions that are not directly parameterized are in green. The dashed line is a reference fit to data reported in literature (Bremer and Dennis^30^). **f** Calculated amino acid uptake rates in the model compared to the maximum uptake rate observed during a growth time series in literature (Zampieri et al.^31^).

We next sought to assess the expanded *E. coli* model by comparing our simulation output first with the original model and then with experimental data. To compare models, we simulated the growth of a cell in rich media, then in minimal glucose media, and finally back in rich media, from many starting cells and over many generations. Comparing time course data for selected model outputs from the original (Fig. 2a) with the new (Fig. 2b) simulations reveals significantly richer dynamics in the new model, in addition to increased fluctuations over time, as compared to relatively constant values in the old version. These fluctuations are the result of increased mechanistic detail in the equations describing regulatory control and the kinetics of cellular processes (metabolism, transcription, and translation), as noted in the previous section. With this additional mechanism in place, rates of production and growth are more closely linked to the stochastic and dynamic internal state of the simulated cell. Specifically, amino acid concentrations now vary over time in response to a variety of factors instead of being constrained to a fixed concentration target (leucine is used as a representative example in Fig. 2a, b). Additionally, charged tRNA concentrations are a new simulation output which plays a role in linking amino acid supply rates with the ribosome elongation rate as well as controlling transcriptional attenuation. More notably, uncharged tRNA concentrations are sensed by RelA and SpoT to provide a dynamic ppGpp concentration. ppGpp controls the relative production of mRNA, rRNA and tRNA, which in turn controls the overall RNAP elongation rate. Additionally, ppGpp binding to RNAP affects the RNAP active fraction and ppGpp inhibition of GTPases associated with the ribosome can limit the ribosome elongation rate. The new simulations thus represent a clear improvement in modeling cellular responses to changing environments, such as the stringent response.

Including the expanded transcriptional regulation and amino acid submodels allows for the simulation of growth under additional environmental conditions, which can be compared to experimental data as a model validation step. The original *E. coli* model simulations exhibited limited growth rate distributions in rich and minimal media conditions, as shown in Fig. 2c. The average growth rates were based on expected growth rates from experimentally characterized conditions, and the variance mainly arose from fluctuations in stochastic initiation and degradation events. Growth rate distributions arising from the new model are shown for four environmental conditions in Fig. 2d, including for two conditions which could not be simulated using the previous version of the model and are not used for parameterization. The corresponding simulation doubling times had wider distributions in the new model and aligned more closely with experimental coefficients of variation (Table 1). The updated model also showed good agreement with other experimental measurements. For example, multiple studies have indicated that a linear trend correlates the growth rate to the RNA:protein ratio (representative of the ribosomal content of cells) across multiple environmental conditions^30^ (Fig. 2e, dashed line). While this correlation was enforced (based on hard-coded mass fractions) in the earlier version of the model, Fig. 2e shows that the trend holds for the new simulations, both in conditions that were used to parameterize the model (rate parameters based on the expected doubling time and mass fractions of RNA and protein) as well as newly predicted conditions that were not directly characterized or used to parameterize the model. Finally, we also compared our simulated amino acid uptake fluxes to the maximum uptake rates reported in literature^31^, and found that they were well-correlated (Fig. 2f). These comparisons therefore strengthened our confidence that the new model was an improvement over the original model, and that it provided an accurate representation of cell growth on a variety of substrates.

**Table 1:** Comparison of growth rates in various media conditions between the model and values reported in literature.

	Previous model			This model			Literature		
Minimal media supplement	Mean	Std	CV	Mean	Std	CV	Mean	Std	CV
+all amino acids	25.8	0.46	0.018	27.5	2.83	0.103	22.5	4.63	0.206
+12 amino acids	−	−	−	31.3	3.19	0.102	26.6	3.79	0.142
+6 amino acids	−	−	−	40.9	7.36	0.180	30.1	4.63	0.154
No amino acids	47.7	1.12	0.023	52.4	7.14	0.136	37.7	5.83	0.155

Mean, standard deviation (Std) and coefficient of variation (CV) for doubling times in minimal glucose media supplemented with various amino acid combinations using default options from the previous model, newly added growth regulation and kinetics (This model) and comparing both to Literature (68). Distributions in doubling times in this model arise inherently from stochastic gene expression and more closely match the CV in literature. Corresponding simulation growth rate distributions are shown in Fig. 2c, d. Simulated results were generated from 432 cell generations (24 initial seeds, 18 generations).

### ppGpp regulation of growth depends on two small molecule feedback inhibition pathways in addition to regulation of gene expression

**Fig. 3:**
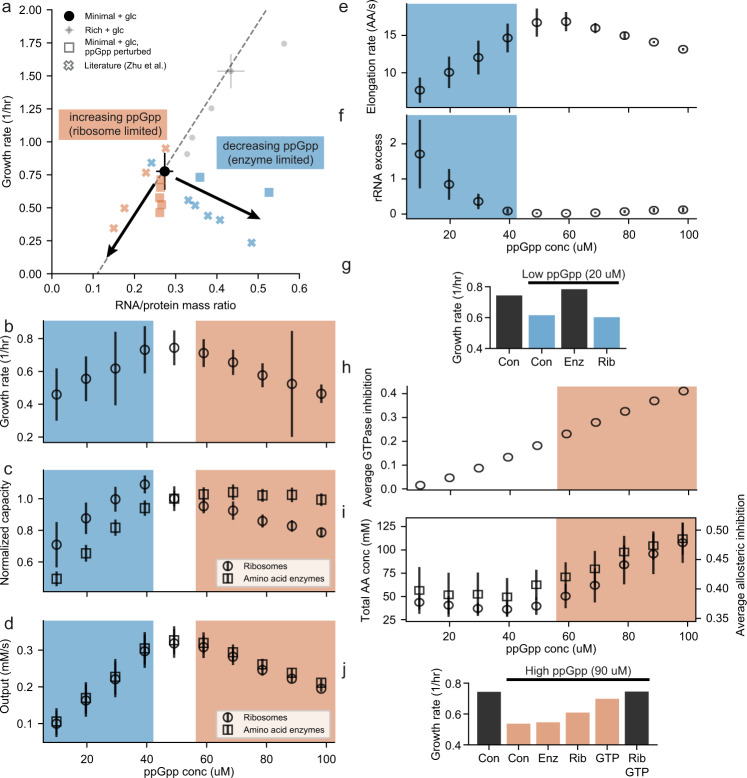
ppGpp regulation of growth depends on two small molecule feedback inhibition pathways in addition to regulation of gene expression. **a** Growth rate vs RNA/protein mass ratio for simulations with perturbed ppGpp concentrations (squares and circles) and literature (crosses and dashed line). Colored points represent minimal glucose media conditions with blue indicating low ppGpp with metabolic enzyme limitations and orange indicating high ppGpp with ribosome limitations. Black arrows indicate the expected trends when ppGpp is perturbed. Average growth rate (**b**), average capacity of ribosomes (circles) or amino acid enzymes (squares) normalized to wildtype capacity (**c**), average total output rates from ribosomes (circles) and amino acid enzymes (squares) (**d**), average elongation rate per ribosome (**e**), and average fraction of ribosome mass that is excess rRNA mass and not included in ribosomes (**f**) from simulations at various concentrations of ppGpp. Blue boxes indicate ppGpp concentrations leading to enzyme limitations while orange boxes represent ppGpp concentrations leading ot ribosome limitations. **g** Average growth rate from simulations at wildtype ppGpp concentrations, low ppGpp concentrations, and low ppGpp concentration with expression modifications (con: control, enz: increased enzyme expression, rib: increased ribosome expression). Average GTPase inhibition by ppGpp (**h**) and total amino acid concentrations (left, circles) leading to an average amino acid pathway allosteric inhibition (right, squares) (**i**) from simulations at various concentrations of ppGpp. **j** Average growth rate from simulations at wildtype ppGpp concentrations, high ppGpp concentrations, and high ppGpp concentration with expression and/or GTPase modifications (con: control, enz: increased enzyme expression, rib: increased ribosome expression, GTP: remove inhibition of GTPases). Simulation data comes from 6 generations and 8 initial seeds (4 initial seeds for **g** and **j**) and error bars represent standard deviation.

With an expanded and validated model in hand, we were ready to tackle key unanswered questions about growth rate control in *E. coli*. For example, the concentration of ppGpp has been modulated experimentally by overexpression of *E. coli* protein RelA to increase ppGpp levels, and *Drosophila melanogaster* protein Mesh 1 to decrease ppGpp levels^32^. One observation from this study seemed paradoxical: that both increasing and decreasing the ppGpp concentration lowered the growth rate, albeit with differing impacts on RNA:protein mass ratios (Fig. 3a). The primary interpretation of these and other data focuses on ppGpp’s control of ribosomal and biosynthetic enzyme expression: at high ppGpp concentrations, the ribosomes become limiting, whereas when ppGpp concentration is decreased, low enzyme expression reins in growth^1,2,32^.

The whole-cell model enables us to assess other factors that could contribute to these observations—for example, product-based allosteric feedback on the amino acid biosynthesis enzymes or translational GTPase inhibition by ppGpp—that could be prohibitively difficult to interrogate experimentally. By comparing experimental observations with simulated outcomes representing elevated or depleted ppGpp concentrations, we hoped to gain deeper insight into the mechanisms that give rise to the physiological outcomes that result from such changes. The *E. coli* model simulations also indicated that an optimal growth rate on glucose minimal media is achieved when ppGpp is set to the wildtype concentration (50 *μ*M), and that both increasing and decreasing the ppGpp concentration will lead to suboptimal growth (Fig. 3a, b). Since this observation was noted in both the simulated and experimental data, we were able to look more deeply into the model to determine the molecular underpinnings of the behavior.

As mentioned above, the “usual suspects” in limiting the growth rate as a result of changes in the ppGpp concentration are ribosomal and biosynthesis enzyme concentrations, which were generally consistent with our simulations. The normalized maximum capacity of ribosomal output was higher than the normalized maximum capacity of enzyme synthesis when ppGpp concentrations were decreased, and conversely, the maximum capacity of amino acid biosynthesis was higher than the ribosomal output when ppGpp levels were increased (Fig. 3c). However, the simulations also produced a more surprising result - namely, that while the maximum possible outputs differed between translation and biosynthesis when ppGpp was increased or decreased, the actual output was matched between the two and lower than for the wildtype ppGpp simulation (Fig. 3d), following the same trends as the growth rate. This suggested that other mechanisms governing the growth rate remained to be identified.

We hypothesized that the reasons for the match were likely to be different for the decreased versus increased ppGpp conditions, and therefore considered each separately. For the case of decreased ppGpp, we found the explanation to be fairly straightforward: lower amounts of amino acid biosynthesis enzymes led to a slower elongation rate per ribosome (Fig. 3e) and thus fewer proteins in general, and r-proteins in particular. We guessed that this could be seen in the free ribosomal RNA concentration in the cell, and in fact there was a distinct increase in free rRNA counts as the ppGpp concentration decreased (Fig. 3f). This hypothesis also suggested that perturbing the biosynthetic enzyme concentrations would enable cells to recover to the optimal growth rate at decreased ppGpp concentrations. Accordingly, we simulated cells experiencing growth at a low ppGpp concentration (20 *μ*M) without any changes, as well as with two possible perturbations: a 25% increase in enzyme expression, or an increase in ribosome expression arising from 50% higher expression of rProtein and 100% higher expression of rRNA. Comparing the growth rates from these simulations to the growth rate expected under optimal ppGpp concentrations (50 *μ*M) showed that increasing enzyme expression rescued to the original growth rate (50 *μ*M ppGpp), while increasing ribosome expression had minimal effect on the growth rate (Fig. 3g), which supported our hypothesis.

The reason for the match between ribosomal and amino acid biosynthetic output at increased ppGpp concentrations was more complex, involving not only ppGpp-dependent transcriptional control of ribosomal biosynthesis, but also two forms of feedback inhibition via small molecule binding. The first form was the higher concentration of ppGpp itself, which impacts the translation rate directly via inhibition of translational GTPases (initiation and elongation factors)^33,34^ (Fig. 3h). With regard to the second form, we noticed that the free amino acid concentrations rose roughly 2.5-fold as we doubled the ppGpp concentration (Fig. 3i, left) because the ribosomal maximum capacity was not sufficient to match the increased enzymatic biosynthesis output. This increase triggered product inhibition of several amino acid biosynthesis pathways (Fig. 3i, right), such that although the enzyme concentration was higher (Fig. 3c), the resulting output was lower (Fig. 3d).

These results suggested a multiple perturbation approach to rescuing the growth rate under increased ppGpp conditions: increasing the ribosome count, which would not only increase translation capacity but also reduce the amino acid pools, leading to less inhibition of the biosynthetic pathways; and eliminating GTPase inhibition, which would lead to a faster elongation rate. In our subsequent simulations, we found that when cellular ppGpp concentrations were high (90 *μ*M), neither increasing the enzyme expression nor the ribosome expression rescued the original growth rate (Fig. 3j). Removing GTPase inhibition was also not sufficient to fully rescue growth. However, removing GTPase inhibition while also increasing the ribosome concentration was observed to completely rescue the growth rate. These results support a more complex understanding of ppGpp’s role in controlling the rate, involving not only ribosomal and enzyme expression, but also the effect of feedback inhibition on translation and amino acid biosynthesis.

### Removing amino acid allosteric inhibition in simulations provides amino acid concentration predictions that are comparable to experimental results

**Fig. 4:**
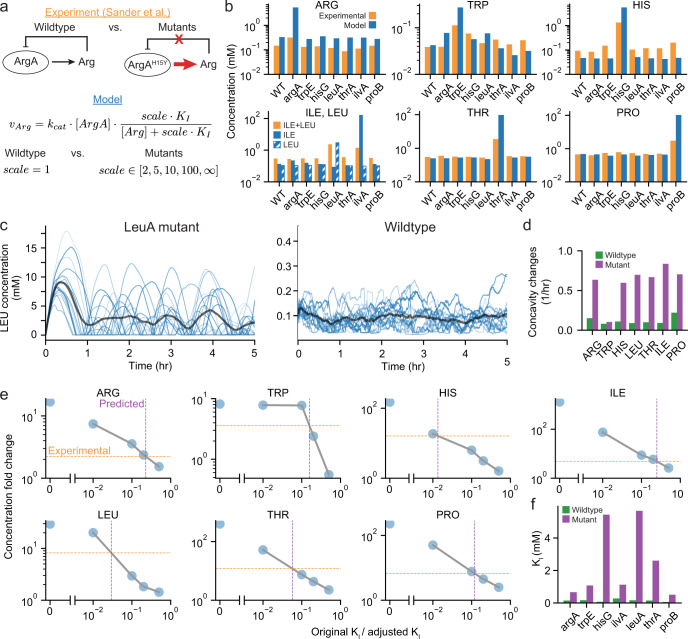
Simulations with amino acid allosteric inhibition removed provide amino acid concentration predictions that are comparable to experimental results. **a** Schematic showing the effect of mutants experimentally, where point mutations remove allosteric inhibition of the amino acid end-product on a pathway enzyme leading to higher amino acid concentrations, and in the model, where the mutation is represented as a scaling factor that increases the end-product inhibitory concentration (*K*_*I*_) that increases the rate of amino acid production. **b** Amino acid concentrations in wild-type cells and mutants with allosteric inhibition removed from experiments (orange, Sander et al.^35^) and the model with scale = infinity (blue). Note that isoleucine and leucine cannot be distinguished experimentally but have distinct concentrations in the model (solid: isoleucine, hatched: leucine). See Supplementary Table 4 for the standard deviation for simulations. **c** Time series for leucine concentrations for LeuA mutant without allosteric inhibition showing oscillatory concentrations without the fast feedback of enzyme inhibition compared to the wildtype with allosteric inhibition. Blue traces show individual cell lineages and the average is shown in gray. **d** Average frequency of concavity changes for each amino acid concentration from wildtype simulations and the mutant simulations corresponding to the amino acid. This is a proxy for 1/period if the fluctuations were regular and periodic. **e** Predictions of the extent of inhibition removal based on the concentration fold changes in experiments compared to simulation fold changes. **f** Allosteric feedback inhibition constants for each enzyme for reported wildtype value (green) and effective *K*_*I*_ in mutants based on analysis in (**d**) (purple). Simulation data comes from 16 generations and 16 initial seeds for (**b**), (**c**), and (**d**) and 8 generations and 4 initial seeds for (**e**) and (**f**).

Since our simulation results highlighted the role of end-product inhibition of amino acid biosynthesis pathways in controlling the growth rate, we next sought to compare the results of allosteric inhibition perturbation experiments with modeling outputs. Experimental results have shown that introducing specific point mutations to synthesis enzymes can remove end-product allosteric inhibition in amino acid synthesis pathways and cause an increase in the concentration of the amino acid product^35^ (Fig. 4a). With the amino acid network kinetics introduced in this newer version of the whole-cell model, we can now simulate mutants that are lacking allosteric inhibition by changing the inhibition parameter, thereby predicting the effect of these mutations on amino acid concentrations (Fig. 4b).

We performed these simulations for mutants in the arginine, tryptophan, histidine, isoleucine, leucine, threonine and proline biosynthetic pathways, and compared average amino acid concentrations after full allosteric removal in the model to the concentrations reported in literature^35^ (Fig. 4b). The comparison showed a qualitative agreement between simulations and experimental results for all pathways, in which the concentration of the amino acid whose inhibition had been abrogated increased, while other amino acids remained at approximately their wildtype levels. This agreement therefore supported both the model and the prior experimental results.

Our comparison was to results that were aggregated across a population at a single time point; however, the whole-cell model can provide further granularity, in the form of single-cell time courses of amino acid concentrations in both wildtype and mutant cells. Examining these time courses in more detail led to further insights; for example, we observed recurrent fluctuations, which occurred on the order of about an hour, from the individual time course traces in mutant simulations but not wildtype (Fig. 4c). These observations are most likely due to the faster time scales of allosteric inhibition as compared to those of transcriptional and translational regulation. The regularity of these fluctuations is seen for the amino acid concentrations in other mutants as well with the rate of concavity changes being lower for the wildtype than mutants (Fig. 4d).

We also noted that in all cases, the model overpredicted the amino acid concentration that arises from the removal of allosteric inhibition (Fig. 4b). This overprediction could result from multiple possible mechanisms. A first possibility is that the model’s representation of negative feedback in the wildtype may be stronger than what actually occurs in *E. coli*. Although this could be due to improper parameterization of the amino acid network or inaccurate expression and regulation of enzymes in the model, it could also point to discrepancies in experimental data and curated knowledge of *E. coli*. For example, metabolite concentration measurements made by different groups can span an order of magnitude or more in the published literature (Supplementary Fig. 1), and can span several orders of magnitude during different phases of bacteria growth^36^. This may explain, for example, why the arginine concentration in the wildtype simulation is already higher than the experimental concentration in the ArgA mutant. Additionally, the concentration of threonine in cells harboring the same ThrA S345F mutation used in Sander et al. is also reported at 82.4 mM elsewhere in literature^37^, which is much closer to the prediction of the model (95.2 mM). It is also possible that the mutants do not experience complete inhibition removal, which was shown for ProB^38^.

Another possibility is that our modeling of the perturbation is too strong, ignoring possible redundant feedback loops which could continue to repress amino acid biosynthesis even in the mutant strain. This could be due to additional allosteric inhibition of the amino acid biosynthesis pathways - for example, in separate reaction steps - that is not captured by the model. Searching the literature, we found that multiple pathways contain more than one enzyme targeted by end-product inhibition, including isoleucine (IlvIH)^39^, leucine (TyrB)^40^, threonine (ThrB)^41^, and proline (ProC)^42^. Given this context, the whole-cell model enables us to estimate how much of the amino acid synthesis pathway inhibition is controlled by allosteric inhibition of the mutant enzymes. First, we calculate the increase in amino acid concentrations at varying levels of allosteric inhibition in the model by scaling the *K*_*I*_ parameter. Comparing the resulting fold change in the end product amino acid concentration in literature to the fold changes in the model can provide the expected change in inhibition that would produce identical concentration fold changes experimentally and computationally (Fig. 4e). The model can then predict an effective *K*_*I*_ that accounts for other potential forms of regulation discussed above (Fig. 4f). We found that four of the amino acids—arginine, tryptophan, isoleucine and proline—had effective *K*_*I*_’s that were within one order of magnitude higher than original values; the other three were within two orders of magnitude. As mentioned above, some additional allosteric regulatory pathways are already known; for other amino acids, such as arginine, the model’s output suggests there may be significant regulation in addition to transcription factors like ArgR and allosteric inhibition of ArgA that are not included in the model or curated in literature. Thus, model predictions can both suggest the presence of additional regulation and also back-calculate the specific contributions of other allosteric regulatory pathways as they become known and better characterized.

### Dynamics of cellular responses during environmental shifts are defined by transient responses and resource reallocation

As mentioned above, a major way in which this version of the *E. coli* model is an improvement over all previous whole-cell modeling efforts is the incorporation of mechanistic regulation in response to changing environments, which enables us to more accurately simulate transitions from one environment to another, particularly downshifts that activate the stringent response. Such transitions are a fundamental aspect of *E. coli’s* life cycle, but they can be challenging to characterize experimentally due to their dynamic and transient nature. Thus, we wanted to use the model to explore cell dynamics during environmental shifts to assess responses to changing nutrient conditions.

**Fig. 5:**
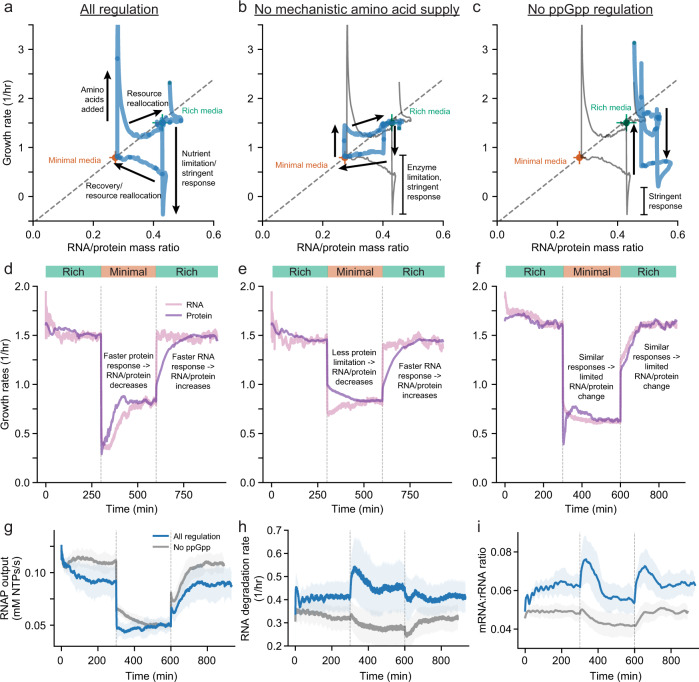
Environmental shifts temporarily deviate from the expected growth rate vs RNA/protein ratio trend as the cell reallocates biomass to optimize growth. Growth rate and RNA/protein mass ratio plotted over time starting in rich media, removing amino acids from the media and adding amino acids back once the cell has adjusted to the new media with all regulation (**a**), no mechanistic amino acid supply (**b**) and no ppGpp regulation (**c**). The blue trace is an average of 32 cell lineages with circles indicating each hour of simulation. The stringent response shows a sharply suppressed growth rate immediately after an environmental downshift with all regulation. Without modeling kinetic amino acid supply, translation is not as limited so the full stringent response will not be activated. Without ppGpp regulation, the cellular composition has limited reorganization because there is not a differential response between the RNA and protein growth rates and the effect of the stringent response can be seen in the difference in growth rate response when compared to having ppGpp regulation included. Growth rate of RNA (light purple) and protein (dark purple) fractions of the cell over time are shown for all regulation (**d**), no mechanistic amino acid supply (**e**) and no ppGpp regulation (**f**). A higher protein growth rate will result in a decreasing RNA/protein ratio, while a higher RNA growth rate will result in an increasing RNA/protein ratio. RNA polymerase output (**g**), RNA degradation rate (**h**), and mRNA to rRNA mass ratio (**i**) for simulations with all regulation included (blue) compared to simulations with no ppGpp regulation (gray). Data comes from 28 generations and 32 initial seeds.

Specifically, we sought to quantify the effects of first removing, and then re-adding, amino acids to a medium in which glucose was the primary carbon source. We simulated this experiment with our model and plotted the resulting trajectory of growth rate versus RNA:protein ratio as we did for condition averages before. This plot presented as a cycle, anchored by stable growth on either the glucose + amino acid (upper right) or glucose minimal (lower left) media, both of which fell on the linear trend correlating the growth rate to the RNA:protein ratio^30^ (see also Fig. 2e, dashed line), with notable transient deviations from the established trend as the simulations reacted to the change in media conditions. In particular, when amino acids are removed from the media, growth is immediately and sharply reduced, but recovers over the next 3 h as the cell reallocates biomass towards a higher protein fraction (Fig. 5a). Similarly, a sharp, transient increase in nutrient uptake and growth rate follows the addition of amino acids to glucose minimal media consistent with single-cell observations^43,44^; this is also followed by a reallocation of resources phase, which lasts approximately 2 h as the cell adjusts to richer media with a higher RNA fraction. For both shifts, the reallocation of resources to biomass components like RNA and protein is not as immediate as the change in growth rate. This is because RNA and protein are generally stable, and thus changes in their concentrations operate on longer time scales than concentrations of small molecules, such as water and amino acids.

We noted with interest that the reallocation from a downshift follows a similar path as ppGpp perturbations that resulted in enzyme limitations (note the points in blue shown in Fig. 3a), while reallocation following an upshift mainly follows the expected trend line after an initial growth rate spike from rapid amino acid uptake. To investigate the downshift in more detail, we re-introduced a component of our earlier *E. coli* model, which did not include kinetic parameters for amino acid biosynthesis and transport (see Fig. 1b bottom left), and instead represented the amount of amino acids supplied to translation as a constant depending on the media condition. Simulated environmental shifts for this perturbation show limited growth rate responses to shifts (Fig. 5b). This suggests that the stringent response during sudden exposure to minimal media depends on enzyme limitation, primarily via regulation of transcription.

To better characterize the role of ppGpp in this cycle, we again simulated the same environmental shifts, but where ppGpp regulation had been removed (Fig. 5c). This simulation output shows limited changes in the RNA/protein ratio, as well as an inability to reallocate resources to obtain an RNA/protein ratio that is optimal for growth on minimal media, supporting prior reports underscoring the central role of ppGpp in setting the RNA:protein ratio^18^.

To further elucidate the mechanism of the shift response, we considered the growth rates of both the RNA and protein separately as they changed over time. In the wildtype case, both RNA and protein growth rates are initially depressed below the expected growth rate for minimal media in response to a downshift in nutrient availability (Fig. 5d). The protein growth rate is able to recover faster, which drives the cell to a lower RNA/protein ratio. This is likely due to dynamics of ppGpp sensing and control: ppGpp senses the charging state of the cell, so the concentration of ppGpp reaches a new steady state once the protein growth rate becomes stable, while the RNA growth rate depends on ppGpp regulation so it normalizes to the new condition only after ppGpp adjusts. In contrast, there is no overshoot of the new growth rate in response to an upshift in nutrient conditions, and the RNA growth rate increases more quickly than the protein growth rate, which leads to an increase in the RNA/protein ratio to support higher growth rates in the rich media. This is primarily due to the higher reserve capacity for RNA polymerases (i.e., the amount of polymerase which is free for immediate use as the shift occurs) as compared to ribosomes. From the simulations which did not include kinetic parameters for amino acid biosynthesis and transport, we further observed that the growth rate of protein does not experience a drop below the expected growth rate in minimal media after a downshift, while RNA still experiences a slightly depressed growth rate relative to the final rate achieved in minimal media (Fig. 5e). Thus, the stringent response is attenuated, while the RNA-protein resource reallocation phase remains intact. For the perturbed simulation in which ppGpp regulation is removed, the growth rate of RNA does not experience a drop below the expected growth rate in minimal media after a downshift while protein still experiences a briefly depressed growth rate (Fig. 5f). Taken together, these observations highlight the interplay between enzyme- based and ppGpp-based limitations on growth over time in changing media conditions.

To better understand the depressed RNA growth rate observed during downshifts with ppGpp regulation (Fig. 5d), we considered the impact of ppGpp on the output rates of RNA polymerases and degradation rates of RNA in more detail. We observed that, without ppGpp regulation, the output from RNA polymerases (Fig. 5g) generally matches the RNA growth rate seen in Fig. 5f, while the RNA degradation rate is relatively constant (Fig. 5h). However, when ppGpp regulation is included, the RNA polymerase output does not drop to the extent that the growth rate does after a downshift, and after an upshift, the growth rate increases to the new growth rate nearly instantaneously while the RNA polymerase output increases steadily over time (Fig. 5d, g). Moreover, we noticed that the RNA degradation rate increases during the downshift, which leads to a decrease in the overall RNA growth rate, but the rate also decreases during the upshift, which helps support a higher RNA growth rate (Fig. 5h). This difference in degradation rate can be explained by differential expression of mRNA, which has a much shorter half life than stable RNA (i.e., rRNA and tRNA). Thus, the sharp increase in ppGpp following a downshift (Fig. 2b) leads to a high fraction of mRNA being transcribed and degraded, while a decrease in the mRNA fraction following an upshift when ppGpp drops results in a reduced degradation rate.

**Fig. 6:**
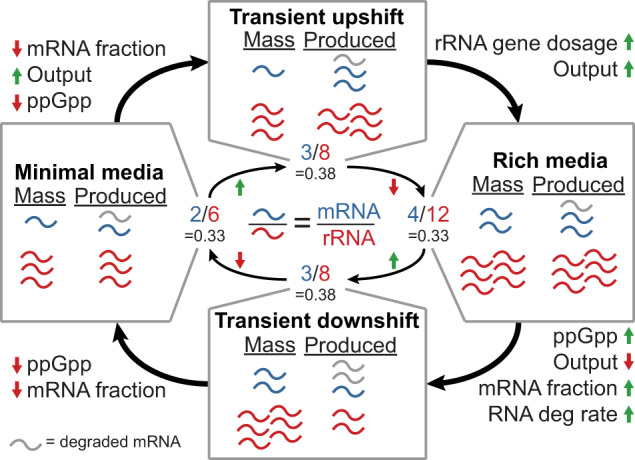
Transient increases in the mRNA to rRNA ratio arise through different mechanisms during nutrient up- or downshifts. Schematic demonstrating a toy example of how an increase or decrease in ppGpp concentration can lead to similar increases in the mRNA to rRNA ratio. “Mass'' represents the amount of RNA (blue: mRNA, red: rRNA, gray: degraded mRNA) that would already be present at the start of a cell cycle and “Produced'' represents the number that will be produced throughout a cell cycle. Note that in minimal or rich media there is balanced growth with mRNA and rRNA both doubling; however, during transient shifts, these amounts need not be balanced. Text in the outer corners describes significant changes when shifting from one box to another based on simulation observations. In the center, the mRNA to rRNA ratio is shown for each condition with the transient shift conditions both showing higher ratios than the steady state minimal or rich media.

Finally, we were surprised to see large but temporary ppGpp-dependent increases in the mRNA to rRNA ratio during both up and downshifts in nutrient conditions (Fig. 5i). These increases appeared similar but occurred for different reasons and with differing mechanisms depending on the shift (Fig. 6). During the initial nutrient downshift, the fraction of newly transcribed RNA that is mRNA increases as higher ppGpp concentrations repress rRNA expression. This leads to a higher production rate of mRNA, and as mRNA concentrations rise, so do the degradation rates. After some time, the initial spike of ppGpp subsides, which leads to a lower fraction of mRNA being produced and lowers the mRNA to rRNA ratio. In contrast, an upshift leads to lower ppGpp concentrations and a decrease in the fraction of mRNA produced, but also an increase in overall RNA polymerase output. This increased output leads to an increase in both mRNA and rRNA production, but has more of an impact on mRNA. This leads to a temporary increase in the mRNA to rRNA ratio until more rRNA is produced as rRNA gene dosage increases along with even higher output at the faster growth rate.

## Discussion

As mentioned in the Introduction, with this work we sought to *explain* some of the key relationships related to growth rate determination from previous studies, as well as to *explore* the complex behaviors and interactions that arise in response to a dramatically changing environment. With regard to explanation, without a comprehensive model, previous studies have focused primarily on interactions between catabolism and transport, protein synthesis, and the size of amino acid pools and the control that can be exerted by or on each. Our model enabled us to incorporate a whole host of other effects, including the coordinated functions of thousands of genes, in ways that not only substantiated the observations and bolstered the conclusions of other labs, but also allowed us to interpret these observations and conclusions in more mechanistic detail. With regard to exploration, we found that a quantitative, holistic, and dynamic view of this organism enabled us to generate several new insights and predictions regarding *E. coli* behavior. Since *E. coli* and other bacteria regularly experience dramatic environmental shifts as part of their oral-fecal life cycles, such findings may be relevant not only to our fundamental understanding of a major model organism, but also to the spread of disease.

From a modeling standpoint, the first and critical point is that the whole-cell model can now calculate growth rate and growth variability based on the internal cellular state represented by the model. Achieving this milestone required several technical advances (detailed above and in the “Methods”) and should unlock new avenues of exploration particularly in areas of cell physiology that depend on growth variability between individual cells, such as persister formation^45^ and survival under stress^46^. However, further modeling advances may be required to enable future progress and a more detailed description of growth. One limitation of the current model is the reduced representation of the amino acid network. In order to parameterize the kinetic reactions to achieve balanced growth in the context of the whole-cell model, reaction pathway kinetics are treated as a single step without considering the accumulation of intermediates, and multiple entry points from central carbon metabolism are ignored. This approach was chosen to reduce the number of unknown parameters that need to be determined and to be able to set up mass balance equations based on the expected supply of amino acids to translation and uptake in rich media in order to solve for those parameters (see “Methods”). Even so, the estimates of these parameters are not unique, and a distribution of a similar set of parameters would likely lead to many of the same phenotypic outcomes we noted in this work, since we already observe similar simulation outcomes from a variety of initial conditions (i.e., different seeds with different internal states). This could be due to regulatory feedback (from either transcriptional regulation and/or small molecule inhibition or saturation), which leads to stability in the simulations despite fluctuations in concentrations, and could also confer robustness to parameter adjustment. Overall, parameterization of large kinetic models of metabolism is a well-known problem with attempts to create scalable methods to determine parameters^47–49^ and while versions of dynamic FBA can provide metabolite dynamics at the genome scale^50^, there are difficulties in constraining kinetics while achieving balanced growth^16^. The approach presented here provides a means of integrating hard kinetic constraints for a subset of metabolism that is fairly well characterized and parameterized and integrating the resulting concentration changes with dynamic FBA all in the context of balanced growth with processes outside of metabolism.

From a biological standpoint, we were surprised to find that without allosteric inhibition of their synthesis pathways, free amino acid concentrations will not only be higher but experience greater variability and even oscillations. This finding has implications when screening single cells such as using FACS for overproduction strain development^51^ where mutants are selected based on single time point measurements that could miss a high production strain that might temporarily be at a low point in the concentration oscillation. It also suggests a critical role of allosteric inhibition in providing stability to internal amino acid pools and confirms the role of allosteric inhibition on the robustness of amino acid biosynthesis^35^. The model demonstrates an important role for mRNA degradation during the stringent response, and also highlights a new phenomenon: that the simulated cell responds similarly to both an upshift and a downshift in nutrient availability with a transient increase in the mRNA:rRNA ratio. Considering this observation from a survival perspective, it seems that producing more mRNA immediately following environmental shifts would allow a cell to adapt to the new environment by shifting expression to take advantage of available resources and match new environmental demands. Whether this phenomenon occurs in response to other environmental shifts (including nutrient shifts, but also temperature, pH or other conditions), as well as whether it can be observed in *E. coli* experimentally, are tantalizing future questions to explore.

Although the new environmental simulations that can now be compared directly to experimental data are encouraging, much remains to be incorporated in order to reach our eventual goal of fully simulating any media condition. For example, our approach to amino acid dynamics here can and should also be extended to better capture the effects of other limitations on growth and ppGpp regulation, including central carbon metabolism, nucleotide synthesis and lipid synthesis^19,52^, and our approach to modeling ppGpp should be applied to other global regulators involved in metabolism, such as determining dynamics of cAMP for improved cAMP-Crp regulation^53^ or expanding Lrp regulatory interactions to capture more Leu-Lrp regulation^54^. Such expansion would likely improve growth rate predictions in a wider range of conditions or metabolic limitations.

Notwithstanding the model’s current prediction capacity, we also noted a number of questions or inconsistencies. One question relates to our observation that the simulated growth rate decreases with either increased or decreased ppGpp, as also shown experimentally^32^ (Fig. 3a). However, the RNA:protein mass ratio trend under perturbed ppGpp conditions showed less consistency between the simulation results and experimental outcomes. Specifically, the ratio appears to be very high at low ppGpp levels, contrary to reported values that appear to approach a value around 0.6^32^, and relatively constant as ppGpp is increased. This suggests the need for additional regulation of rRNA transcription in the model such as through initiating ribonucleotides^55^, or may hint at a more direct (and unannotated) regulatory role of rRNA itself, similar to how ribosomal proteins can regulate their own expression^56^. Similarly, the higher amino acid concentrations simulated for mutants lacking allosteric regulation (see Fig. 4b, e, f), as compared to experiments^35^, also suggest the presence of other regulatory pathways that are not accounted for in the model and perhaps even currently unknown. Answering these questions is worthy of a significant experimental effort in the future.

In sum, our work presents a significant step forward towards achieving a whole-cell model of *E. coli* that not only accounts for the known functionality of genes but also the functionality of small molecules and has the ability to simulate a variety of new environments that *E. coli* might encounter. In the process, it also helps to explain mechanistically how growth rate control operates in responding to changing conditions. We anticipate that this new functionality, and the insights it can catalyze, will support a variety of new applications with and expansions of the *E. coli* Whole-Cell Modeling Project^17^.

## Methods

Here we describe three aspects of the methods used to produce this work. First, although the modeling framework (including the whole-cell modeling approach, model construction and simulation algorithm) has essentially remained the same since the first version of the *E. coli* model described in the supplement of Macklin et al.^16^, additional data preprocessing and submodels have been updated as described in the first section below. Second, we include a detailed list of how all the simulations were run. Third, we describe how outputs from the simulations were used to produce each figure panel. Note that all code used to parameterize the model, run simulations and analyze the output is available on GitHub at https://github.com/CovertLab/WholeCellEcoliRelease.

### Computational and modeling methods

#### Modeling features added

We found that the features described below were sufficient to capture the dynamics of environmental shifts with amino acids and provide a mechanistic determination of the growth rate of the cell in a variety of media conditions. Although whole-cell modeling is made possible by treating processes as independent on short time scales, this project demonstrated that those processes can become heavily dependent on each other. Including dynamic amino acid concentrations was an essential first step that relied on combining amino acid biosynthesis, transport and tRNA charging. Amino acid supply is tightly coupled with tRNA charging and both happen on very fast time scales compared to other model processes so it was necessary to abstract amino acids pathways out of the main metabolism submodel, integrate both amino acid supply and tRNA charging kinetics on sub-timestep scales, and update metabolism with the result. This change led to responsive amino acid concentrations, which provided buffering capacity to tRNA charging to improve stability of that submodel. tRNA charging rates provide a more responsive translation rate to more accurately capture the protein growth rate as a function of the cellular state. Perhaps more importantly, tRNA charging levels are required for proper modeling of ppGpp as RelA and SpoT sense the charging state of the cell and control the concentration of ppGpp. All of the mechanistic processes added are directly controlled by transcriptional regulation (ppGpp, transcription factors and transcriptional attenuation), which will affect expression of the functionally implemented genes. The newly added regulation was crucial to maintain stable amino acid pools and respond to changing environments. Missing or incorrect data for any of these regulatory elements could lead to instability during development and ultimately, required all of the features to be integrated together for all of them to work as expected.

#### Amino acid biosynthesis

Rates of amino acid synthesis play a critical role in limiting the amount of protein that a cell can make by specifying the amount of precursors that can be made available to ribosomes. A newly added simulation option (--mechanistic-translation-supply) uses a kinetic approach to amino acid biosynthesis pathways to determine the rate of supply to translation that is dependent on enzyme expression, amino acid concentrations and the linked network topology of different amino acid pathways. Amino acid biosynthesis pathways display a wide range of complexity from single, reversible reaction steps, to long linear pathways, and even some branching pathways with shared branchpoint intermediates. Mass enters each pathway from various points in metabolism (Supplementary Fig. 2). One common precursor used in all pathways is glutamate. With this in mind we make the assumption that glutamate is upstream of all amino acid pathways and a single step occurs between each amino acid (Supplementary Fig. 2)). Simplifying to a single reaction step starting from glutamate allows for simple parameterization that maintains expected amino acid concentrations and provides enough supply to translation to maintain balanced growth.

Parameters for each amino acid pathway (reaction) are determined before simulations based on the following rate equations:

1 $${v}_{supply,i}={v}_{synth,i}-{v}_{down,i}-{v}_{deg,i}-{v}_{rev,i}+{v}_{exchange,i}$$

2 $${v}_{synth,i}={k}_{cat,f,i}\cdot {E}_{f,i}\cdot \frac{1}{1+\frac{A{A}_{i}}{{K}_{I,i}}}\cdot \mathop{\prod}\limits_{j\in \{u{p}_{i}\}}\frac{1}{1+\frac{{K}_{M,j}}{A{A}_{j}}}$$

3 $${v}_{down,i}=\mathop{\sum}\limits_{j\in \{dow{n}_{i}\}}{v}_{synth,j}$$

4 $${v}_{deg,i}={k}_{cat,deg,i}\cdot {E}_{deg,i}\cdot \frac{1}{1+\frac{{K}_{M,deg,i}}{A{A}_{i}}}$$

5 $${v}_{rev,i}={k}_{cat,rev,i}\cdot {E}_{rev,i}\cdot \frac{1}{1+\frac{{K}_{M,rev,i}}{A{A}_{i}}}$$

where *v*_*supply*,*i*_ is the rate of amino acid supply to translation in a given time step and determined by *v*_*synth*,*i*_, *v*_*down*,*i*_, *v*_*deg*,*i*_, *v*_*rev*,*i*_, *v*_*import*,*i*_, *v*_*exchange*,*i*_, which are rates of synthesis, loss to downstream amino acids, degradation loss, loss to reverse reactions, exchange with the environment, respectively, for amino acid, *i*. Not all rates, such as reverse or degradation rates, exist for each amino acid depending on the network topology specified in Supplementary Fig. 2. Furthermore, for a given amino acid, only a reverse or degradation reaction can be present in our representation since we can only solve for two *k*_*cat*_ parameters (forward and loss) given the constraints described below. *k*_*cat*_ is the reaction rate constant, *E* is the enzyme concentration, *AA* is the amino acid concentration, *K*_*I*_ is the allosteric inhibition constant, {*up*} and {*down*} are the sets of amino acids that are upstream or downstream of the given amino acid, and *K*_*M*_ is the Michaelis constant.

Enzyme concentrations (*E*) come from expected expression in a given environmental condition. Enzymes capable of catalyzing the same reaction step are summed together and given the same parameters to simplify the parameter calculation. Amino acid concentrations (*AA*_*i*_) come from expected concentrations in a given environmental condition.

*K*_*I*_ parameters are determined based on reported values in literature and the expected amino acid concentration in minimal media such that the *K*_*I*_ is as close to the concentration while staying within the bounds of reported data (see Supplementary Table 2).

*K*_*M*_ parameters for upstream amino acids, reverse reactions and degradation reactions come from various literature sources. In cases where data is not available, we use the following assumptions in order to get saturating kinetics: upstream reaction *K*_*M*_ are assumed to be the amino acid concentration in minimal media for a high dynamic range while reverse and degradation *K*_*M*_ values are assumed to be 10 times the amino acid concentration in minimal media in order to have limited loss at physiological concentrations (see Supplementary Table 2).

We use an optimization problem to solve for *k*_*cat*_ parameters and environmental exchange rates (*v*_*exchange*_) using concentrations in minimal glucose media and rich media with all amino acids added. At steady-state amino acid concentrations, a mass balance around each amino acid links metabolic rates (synthesis and transport) to translation, which has a known rate—the rate required to meet protein doubling demands for the two growth conditions. Along with known rates of transport from literature^31^, we should be able to solve for the remaining two unknowns (forward and loss *k*_*cat*_) with two equations (minimal and rich media):

6 $${k}_{cat,f}\cdot {C}_{synth,minimal}-{k}_{cat,r}\cdot {C}_{loss,minimal}={S}_{minimal}+{v}_{down,minimal}$$

7 $${k}_{cat,f}\cdot {C}_{synth,rich}-{k}_{cat,r}\cdot {C}_{loss,rich}={S}_{rich}+{v}_{down,rich}-{v}_{exchange}$$

where *C* is the capacity of a forward or reverse reaction and defined as the enzyme concentration multiplied by saturation terms or $C=\frac{v}{{k}_{cat}}$, *S* is the rate of supply of amino acids to translation needed to double the protein mass within a cell cycle, and *C*_*loss*_ represents the capacity for either the reverse, *v*_*rev*_, or degradation, *v*_*deg*_ rate.

However, the additional constraint of having positive *k*_*cat*_ parameters makes solving the set of equations infeasible for some amino acids. For this reason, we must use an optimization approach. Varying the uptake rate is sufficient to provide a non-negative solution for the *k*_*cat*_ parameters. Setting an objective function to closely match the available data for *k*_*cat*_ values and uptake rates allows us to select a set of parameters from the possible solution space:

8 $$objective=1000\cdot | | {v}_{exchange}-{v}_{exchange,lit}| | +| | {k}_{cat}-{k}_{cat,lit}| | +| | {k}_{cat,rev}| |$$

#### Amino acid transport

Transport of amino acids from the environment provides additional nutrient sources for the cell and reduces the amount of energy and resources that need to be spent on amino acid biosynthesis as well as the translation of enzymes to catalyze biosynthesis. Together, this helps support higher growth rates in rich media containing amino acids. From the optimization in Eq. (8), we can get exchange rates that can be used to parameterize transport of amino acids based on concentrations of transporters and amino acids. Using the new --mechanistic-aa-transport simulation option, the simulation will calculate an exchange rate based on Eq. (9) when a given amino acid is in the media, otherwise the rate will be a constant rate based on the value from Eq. (8) when amino acids are present. We assume that the rate of uptake is inhibited by internal amino acid concentrations as is the case with many transporters^57,58^ and that export of each amino acid occurs with Michaelis–Menten kinetics:

9 $${v}_{exchange,i}={v}_{import,i}-{v}_{export,i}$$

10 $${v}_{import,i}={k}_{cat,im,i}\cdot {T}_{im,i}\cdot \frac{1}{1+\frac{A{A}_{i}}{{K}_{I,i}}}$$

11 $${v}_{export,i}={k}_{cat,ex,i}\cdot {T}_{ex,i}\cdot \frac{1}{1+\frac{{K}_{M,i}}{A{A}_{i}}}$$

where *v* represents rates of transport, *k*_*cat*_ is the rate constant for import or export, *T* is the transporter expression for import or export, *AA* is the amino acid concentration, *K*_*I*_ is the inhibition constant for import, and *K*_*M*_ is the Michaelis constant for export for each amino acid, *i*.

Transporter to amino acid mappings come from annotated function on EcoCyc^29^. If multiple transporters exist for a given amino acid, the transporters are summed together and given the same set of parameters. All amino acids can be imported in the model except for cysteine which does not have a dedicated importer^29,59^ and shows minimal uptake from the environment ^31^.

*K*_*I*_ values are assumed to be the expected concentration of each amino acid in rich media.

Some *K*_*M*_ values come from literature and are based on steady-state internal concentrations of amino acids when corresponding dipeptides are added to the external media, which literature suggests should be near the *K*_*M*_^60^ (see Supplementary Table 3). For those that do not have data available, a *K*_*M*_ is assumed based on the average increase of the other amino acid *K*_*M*_ values over the amino acid concentration in minimal media:

12 $$factor=\frac{{\sum }_{i}\frac{{K}_{M,i}}{A{A}_{i}}}{n}$$

where *i* is each amino acid with data and *n* is the number of amino acids with data so that unknown *K*_*M*_ values can be estimated:

13 $${K}_{M,j}=factor\cdot A{A}_{j}$$

for each amino acid, *j*, without data.

To solve for *k*_*cat*_ parameters for import and export, we use the relationship in Eq. (9) under two conditions: when amino acids concentrations are at the expected concentrations in rich media, the exchange rate will be the rate calculated from Eq. (8) and the assumption that when amino acid concentrations are equal to the export *K*_*M*_, *v*_*exchange*_ will be 0, which is how the *K*_*M*_ values were defined when curated.

This approach does not take into account competition of multiple amino acids in the media for transporters that can recognize multiple amino acids. For simplicity in solving for the *k*_*cat*_ parameters based on the available equations, we also are unable to treat multiple transporters for each amino acid separately even though *k*_*cat*_, *K*_*I*_, and *K*_*M*_ parameters would likely vary for each transporter.

#### Phenomenological amino acid supply

This option provides an alternative to mechanistic amino acid biosynthesis and transport and is not dependent on the expression of enzymes and transporters but is responsive to amino acid concentrations to help provide stability to tRNA charging. This approach is not used in simulations with all regulation but instead when mechanistic amino acid supply is turned off (--no-mechanistic-aa-supply). With this option, the model uses a phenomenological approach to determining the supply rate instead of relying on the kinetics described above:

14 $${v}_{supply,i}={f}_{supply,i}\cdot {v}_{supply,i,media}$$

where *v*_*supply*,*i*,*media*_ is calculated before simulations for each media condition with a known doubling time based on the rate needed to double the protein fraction and *f*_*supply*,*i*_ is defined as:

15 $${f}_{supply,i}={f}_{base{{{\_}}}synthesis,i}+{f}_{inhibited{{{\_}}}synthesis,i}+{f}_{import,i}-{f}_{export,i}$$

where

16 $${f}_{base\_synthesis,i}={c}_{1,i}$$

17 $${f}_{inhibited\_synthesis,i}=\frac{1}{1+\frac{[A{A}_{i}]}{{K}_{I,i}}}$$

18 $${f}_{import,i}=\left\{\begin{array}{ll}{c}_{2,i}\quad &{{{\rm{if}}}}\,A{A}_{i}\,{{{\rm{in}}}}\,{{{\rm{environment}}}}\\ 0\quad &{{{\rm{otherwise}}}}\end{array}\right.$$

19 $${f}_{export,i}=\frac{[A{A}_{i}]}{{K}_{M,i}+[A{A}_{i}]}$$

*c*_1,*i*_, *c*_2,*i*_, *K*_*I*,*I*_, and *K*_*M*,*i*_ can be determined by defining parameters *f*_*I*_ and *f*_*M*_ and constraints below that represent the fraction of contributions to the supply rate at the expected amino acid concentrations when the cell is in the presence of amino acids in the environment and when amino acids are not present:when [*AA*_*i*_] = [*AA*_*i,basal*_]:

20 $${f}_{inhibited\_synthesis,i}={f}_{I}$$

21 $${f}_{supply,i,basal}=1$$

when [*AA*_*i*_] = [*AA*_*i,amino acid*_]:

22 $${f}_{export,i}={f}_{M}$$

23 $${f}_{supply,i,amino\_acid}=1$$

Solving shows that the parameters are defined as

24 $${K}_{I,i}=\frac{{f}_{I}\cdot [A{A}_{i,basal}]}{1-{f}_{I}}$$

25 $${K}_{M,i}=\left(\frac{1}{{f}_{M}}-1\right)\cdot [A{A}_{i,amino\_acid}]$$

26 $${c}_{1,i}=1-\left({f}_{I}-\frac{[A{A}_{i,basal}]}{{K}_{M,i}+[A{A}_{i,basal}]}\right)$$

27 $${c}_{2,i}=1-\left({c}_{1,i}+\frac{1}{1+\frac{[A{A}_{i,amino\_acid}]}{{K}_{I,i}}}-{f}_{M}\right)$$

This approach does not depend on the expression of enzymes or transporters and does not take the topological amino acid synthesis network and precursors into account. The base rate is assumed to change immediately upon a change in the environment without accounting for the time it takes for regulation to alter expression. Terms are dependent on the amino acid concentration to provide stability and provide an approximation of the control amino acid concentrations can have over the rates by increasing the supply at low amino acid concentrations and decreasing the supply at high amino acid concentrations.

#### tRNA charging

tRNA charging plays a central role in linking amino acid kinetics to translation (and ultimately the overall cellular growth rate). Additionally, new transcriptional regulatory features are dependent on tRNA charging - ppGpp concentrations indicate levels of uncharged tRNA in the cell and charged tRNA concentrations affect transcriptional attenuation. In the model, tRNA charging is used in PolypeptideElongation to capture a more mechanistic view of translation but can be optionally disabled with a simulation option (--no-trna-charging). With no tRNA charging, translation is simply limited by the codon sequences actively translated mRNAs and a constant rate of amino acid supply that is condition dependent based on the expected doubling time. With tRNA charging, the rate of amino acid incorporation becomes a function of the state of the cell including the codon sequence of mRNAs being translated as well as amino acid, tRNA, synthetase and ribosome concentrations. With the assumption that charging happens sufficiently fast (*k*_*cat*_ ≈ 100 s^−1^ vs ~1 s time step) and the state of the cell does not significantly change between time steps, the ratio of uncharged to charged tRNA can be adjusted until rates of tRNA charging (*v*_*charging*_) and ribosome elongation (*v*_*elongation*_) reach a steady state during each time step. This is shown with ODEs for each tRNA species, *i*, shown below:

28 $$\frac{{\rm{d}}[tRN{A}_{charged,i}]}{{\rm{d}}t}={v}_{charging,i}-{v}_{elongation,i}$$

29 $$\frac{{\rm{d}}[tRN{A}_{uncharged,i}]}{{\rm{d}}t}=-\frac{{\rm{d}}[tRN{A}_{charged,i}]}{{\rm{d}}t}$$

The rates of charging and elongation are based on previous work ^9^ and defined below:

30 $${v}_{charging,i}={k}_{S}\cdot [synthetas{e}_{i}]\cdot \frac{\frac{[tRN{A}_{uncharged,i}]}{{K}_{M,tRN{A}_{u},i}}\cdot \frac{[A{A}_{i}]}{\cdot {K}_{M,aa,i}}}{1+\frac{[tRN{A}_{uncharged,i}]}{{K}_{M,tRN{A}_{u},i}}+\frac{[A{A}_{i}]}{{K}_{M,aa,i}}+\frac{[tRN{A}_{uncharged,i}]}{{K}_{M,tRN{A}_{u},i}}\cdot \frac{[A{A}_{i}]}{\cdot {K}_{M,aa,i}}}$$

31 $${v}_{elongation,i}={f}_{i}\cdot \frac{{k}_{rib}\cdot [ribosome]}{1+{\sum }_{j}\left({f}_{j}\cdot \left(\frac{{K}_{D,tRN{A}_{c}}}{[tRN{A}_{charged,j}]}+\frac{[tRN{A}_{uncharged,j}]}{[tRN{A}_{charged,j}]}\cdot \frac{{K}_{D,tRN{A}_{c}}}{{K}_{D,tRN{A}_{u}}}\right)\right)}$$

where *k*_*S*_ is the synthetase charging rate, ${K}_{M,tRN{A}_{u}}$ is the Michaelis constant for free tRNA binding to synthetases, *K*_*M*,*aa*_ is the Michaelis constant for amino acids binding synthetases, *f*_*i*_ is the fraction of codon *i* to total codons to be elongated, *k*_*rib*_ is the max ribosome elongation rate, ${K}_{D,tRN{A}_{c}}$ is the dissociation constant of charged tRNA to ribosomes and ${K}_{D,tRN{A}_{u}}$ is the dissociation constant of uncharged tRNA to ribosomes. *K*_*M*_ values for tRNA and amino acids come from literature with default values of 1 *μ*M and 100 *μ*M, respectively, for species that do not have curated data^9^. Charging currently ignores the concentration of ATP in determining the rate.

*k*_*rib*_ from Eq. (31) can be defined in two ways depending on the simulation options that are set. With --no-ppgpp-regulation or --disable-ppgpp-elongation-inhibition selected, *k*_*rib*_ will be a constant. Otherwise, when --ppgpp-regulation is selected, *k*_*rib*_ is determined based on the current ppGpp concentration and competitive inhibition of GTPases associated with translation (initiation and elongation factors):

32 $${k}_{rib}=\frac{{k}_{rib,max}}{1+{\left(\frac{ppGpp}{{K}_{I}}\right)}^{H}}$$

where *k*_*rib*,*max*_ is the maximum elongation rate, *K*_*I*_ is the inhibition constant and *H* is a Hill coefficient reflecting cooperativity. The parameters are fit with a least squares approach using ppGpp concentrations and elongation rates from literature^30^.

With tRNA charging, translation will be limited by the calculated elongation rate (*v*_*elongation*_) instead of the supply of amino acids to PolypeptideElongation. With a variable amount of amino acids being produced and used at each time step, the concentration of each amino acid species, *i*, in the cell can vary as shown below, which will update the homeostatic target in Metabolism:

33 $$\frac{{\rm{d}}[A{A}_{i}]}{{\rm{d}}t}={v}_{supply,i}-{v}_{charging,i}$$

where *v*_*supply*,*i*_ is the rate of supply of amino acids from Eq. (1) or Eq. (14), which includes both synthesis and uptake, and *v*_*charging*,*i*_ is the rate of charging as determined above.

When the --mechanistic-translation-supply option is used in simulations, tRNA charging can show some instability due to the assumption that the amino acid concentration remains constant throughout the timestep as charging occurs. To improve stability, the --aa-supply-in-charging option should also be used. This option allows the amino acid concentration used in the charging ODEs (Eq. (28), Eq. (29)) to vary on sub-timestep scales based on the ODEs defined with amino acid supply (Eq. (33)).

#### ppGpp kinetics

As a major regulator of growth in bacteria^18^, including ppGpp dynamics in a model designed to capture growth and environmental responses is imperative. Through RelA, ppGpp synthesis occurs in coordination with translation at the ribosome so reactions are modeled together within the PolypeptideElongation process. Reactions are only modeled when two simulation options are enabled together (--trna-charging and --ppgpp-regulation). In addition to RelA, SpoT is also responsible for producing ppGpp as well as hydrolyzing ppGpp so the total change in ppGpp concentration can be written as:

34 $$\frac{{\rm{d}}{C}_{ppGpp}}{{\rm{d}}t}={v}_{RelA}+{v}_{SpoT,syn}-{v}_{SpoT,deg}$$

where *v*_*RelA*_ is the rate of ppGpp production by RelA, *v*_*SpoT*,*syn*_ is the rate of ppGpp production by SpoT and *v*_*SpoT*,*deg*_ is the rate of degradation of ppGpp by SpoT. The equations that govern these rates are shown below:

35 $${v}_{RelA}={k}_{RelA}\cdot {C}_{RelA}\cdot \frac{\frac{{C}_{rib:tRN{A}_{u,i}}}{{K}_{D,RelA,i}}}{\frac{{C}_{rib:tRN{A}_{u,i}}}{{K}_{D,RelA,i}}+{\prod }_{j\ne i}\left(1+\frac{{C}_{rib:tRN{A}_{u,j}}}{{K}_{D,RelA,j}}\right)}$$

36 $${v}_{SpoT,syn}={k}_{SpoT,syn}\cdot {C}_{SpoT}$$

37 $${v}_{SpoT,deg}={k}_{SpoT,deg}\cdot {C}_{SpoT}\cdot {C}_{ppGpp}\cdot \frac{1}{1+{\sum }_{i}\frac{{C}_{tRN{A}_{u,i}}}{{K}_{I,SpoT,i}}}$$

where *k*_*RelA*_, *k*_*SpoT*,*syn*_, and *k*_*SpoT*,*deg*_ are rate constants for ppGpp production by RelA, ppGpp production by SpoT and ppGpp degradation by SpoT, respectively, *C*_*RelA*_, ${C}_{rib:tRN{A}_{u}}$, *C*_*SpoT*_, *C*_*ppGpp*_, and ${C}_{tRN{A}_{u}}$ are concentrations for RelA, ribosomes bound to uncharged tRNA, SpoT, ppGpp and uncharged tRNA, respectively, *K*_*D*,*RelA*,*i*_ is a dissociation constant for RelA binding ribosomes for each uncharged tRNA species at the A-site and *K*_*I*,*SpoT*,*i*_ is an inhibition constant for the effect of each species of uncharged tRNA on SpoT mediated degradation of ppGpp. *v*_*RelA*_ includes an adjustment for the competitive inhibition for all other tRNA species that RelA could recognize represented by the product. Parameters for *K*_*D*,*RelA*,*i*_ are based on those reported in literature^9^. Direct inhibition of uncharged tRNA on SpoT hydrolase activity is assumed based on reports in literature^61,62^ with the base *K*_*I*,*SpoT*,*i*_ parameter being assumed such that the value is sufficiently high to be above concentrations of uncharged tRNA that are normally present during exponential growth at different rates^61^ while also empirically providing qualitatively stable ppGpp concentrations in simulations. Both *K*_*D*,*RelA*,*i*_ and *K*_*I*,*SpoT*,*i*_ are adjusted by the prevalence of each tRNA species, which gives each tRNA species approximately the same amount of control over ppGpp production and degradation. This is based on reports in the literature that show different tRNA species can have varying effects on ppGpp production^63^. The adjustment to both parameters is calculated prior to simulations based on expected total tRNA concentrations (*C*_*tRNA*_):

38 $$rati{o}_{i}=\frac{{C}_{tRN{A}_{i}}}{{\sum }_{j}{C}_{tRN{A}_{j}}}$$

39 $$adjustmen{t}_{i}=\frac{rati{o}_{i}}{\frac{{\sum }_{j}rati{o}_{j}}{{n}_{tRNA}}}$$

40 $${K}_{D,RelA,i}=adjustmen{t}_{i}\cdot {K}_{D,RelA,lit}$$

41 $${K}_{I,SpoT,i}=adjustmen{t}_{i}\cdot {K}_{I,SpoT,lit}$$

where *n*_*tRNA*_ is the number of tRNA species

Concentrations for all species necessary for calculations in Eq. (34) except for ${C}_{rib:tRN{A}_{u}}$ are directly tracked by the model. With Eq. (31), the concentration of ribosomes bound to uncharged tRNA can be calculated for each species, *i* as follows:

42 $${C}_{rib:tRN{A}_{u,i}}={C}_{rib,i}\frac{\frac{{C}_{tRN{A}_{u},i}}{{K}_{D,tRN{A}_{u}}}}{1+\frac{{C}_{tRN{A}_{u},i}}{{K}_{D,tRN{A}_{u}}}+\frac{{C}_{tRN{A}_{c},i}}{{K}_{D,tRN{A}_{c}}}}$$

where *C*_*rib*,*i*_ is the concentration of ribosomes with species *i* at the A-site and defined as

43 $${C}_{rib,i}=\frac{{v}_{elongation,i}}{{\sigma }_{i}\cdot {k}_{rib}}$$

where *v*_*elongation*,*i*_ is defined in Eq. (31), *k*_*rib*_ is the max ribosome elongation rate and *σ*_*i*_ is A-site fraction saturated with charged tRNA defined as

44 $${\sigma }_{i}=\frac{\frac{{C}_{tRN{A}_{c},i}}{{K}_{D,tRN{A}_{c}}}}{1+\frac{{C}_{tRN{A}_{u},i}}{{K}_{D,tRN{A}_{u}}}+\frac{{C}_{tRN{A}_{c},i}}{{K}_{D,tRN{A}_{c}}}}$$

SpoT degradation inhibition by uncharged tRNA is based on work by Murray and Bremer^61^. Parameters for SpoT are also calculated with data from that work with the assumption that SpoT is at a concentration of 0.1 *μ*M. With a ppGpp half life of 30 s with no ppGpp synthesis and inhibited translation (assume fully charged tRNA and no uncharged tRNA to inhibit ppGpp degradation), *k*_*SpoT*,*deg*_ can be determined by integrating the following ODE:

45 $$\frac{{\rm{d}}{C}_{ppGpp}}{{\rm{d}}t}=-{k}_{SpoT,deg}\cdot {C}_{SpoT}\cdot {C}_{ppGpp}$$

which gives the following solution with the measured half life:

46 $${k}_{SpoT,deg}=\frac{\ln (2)}{{t}_{1/2}}\cdot \frac{1}{{C}_{SpoT}}=\frac{\ln (2)}{30}\cdot \frac{1}{0.1}=0.23\frac{1}{\mu M\cdot s}$$

This value can then be used to solve for the synthesis rate constant by using the measured ppGpp concentration (6 pmol/OD from Murray and Bremer or 11.4 *μ*M with OD to volume conversion for an average cell) and assuming a steady state concentration of ppGpp in a RelA knockout with no degradation inhibition:

47 $$\frac{{\rm{d}}{C}_{ppGpp}}{{\rm{d}}t}={k}_{SpoT,syn}\cdot {C}_{SpoT}-{k}_{SpoT,deg}\cdot {C}_{SpoT}\cdot {C}_{ppGpp}=0$$

48 $${k}_{SpoT,syn}={k}_{SpoT,deg}\cdot {C}_{ppGpp,SS}$$

Solving for *k*_*SpoT*,*syn*_:

49 $${k}_{SpoT,syn}=0.23\cdot 11.4=2.6\,{{\rm{s}}}^{-1}$$

#### ppGpp regulation

Although ppGpp has many effects on physiology throughout the cell, interactions with RNAP and control of transcription is one of the main ways it helps the cell respond to the environment and optimize growth. This relies on dynamics of ppGpp as described in the previous section and exhibits regulatory control over many of the features described here through downregulation of ribosomes (rRNA and rProtein), tRNA, tRNA synthetases, RNAP, SpoT and other genes as well as upregulation of amino acid synthesis enzymes and RelA.

Without ppGpp regulation, the probability of initiating a transcript is defined as

50 $${v}_{synth,j}={\alpha }_{j}+\mathop{\sum}\limits_{i}{P}_{T,i}{{\Delta }}{r}_{i,j}$$

where *α*_*j*_ represents basal recruitment of RNA polymerase, *P*_*T*,*i*_ is the probability that the *i*^*th*^ transcription factor is DNA-bound and Δ*r*_*i*,*j*_ is the recruitment effect of the *i*^*th*^ transcription factor on the *j*^*th*^ gene.

ppGpp regulation of gene expression can be enabled with a simulation option (--ppgpp-regulation). When ppGpp regulation is enabled, Eq. (50) must be modified to account for the effect of ppGpp with the main difference being that the basal recruitment rate (*α*_*j*_) is now dependent on ppGpp. With a variable *α*_*j*_, we must also adjust the recruitment effect of transcription factors (Δ*r*) to prevent negative or 0 synthesis probabilities for many genes that are regulated by both ppGpp and transcription factors. This results in a slightly adjusted form of Eq. (50):

51 $${v}_{synth,j}={\alpha }_{j}+\frac{{\alpha }_{j}}{{\alpha }_{j,o}}\cdot \mathop{\sum}\limits_{i}{P}_{T,i}{{\Delta }}{r}_{i,j}$$

where *α*_*j*,*o*_ is the basal probability used to calculate the original recruitment parameters so that transcription factors will always reduce expression by the same fraction regardless of the basal probability calculated by the current ppGpp concentration.

To calculate parameters for the new equation, we start by assuming that ppGpp binds to RNA polymerases and the free and bound forms of RNA polymerase have different amounts of expression for each gene. The binding can be represented with a reversible reaction:

52 $$RNA{P}_{free}+ppGpp\rightleftharpoons RNA{P}_{ppGpp}$$

To calculate the impact of ppGpp on RNA expression the following equation is used:

53 $$ex{p}_{j}=(1-f)\cdot ex{p}_{free,j}+f\cdot ex{p}_{ppGpp,j}$$

where *exp*_*free*,*j*_ and *exp*_*ppGpp*,*j*_ represent the amounts of expression expected from the free and ppGpp bound RNA polymerases, respectively, and are determined prior to simulations. *f* is the fraction of RNA polymerases that are bound to ppGpp as defined below:

54 $$f=\frac{RNA{P}_{ppGpp}}{RNA{P}_{total}}=\frac{{C}_{ppGpp}^{2}}{{K}_{M}^{2}+{C}_{ppGpp}^{2}}$$

where *C*_*ppGpp*_ is the concentration of ppGpp, *K*_*M*_ is determined prior to simulations and represents the Michaelis constant representing the concentration of ppGpp where half the RNA polymerases are bound. There is also a Hill coefficient of 2 representing the fact that ppGpp has multiple sites of interaction with RNA polymerase^64^.

To calculate *K*_*M*_ of ppGpp binding to RNA polymerase, several assumptions are made. The first is that the amount of RNA in a cell at different growth rates changes due to changes in stable RNA expression. The second is that this change in RNA is controlled by ppGpp concentration changes which cause differential expression based on the amount of RNA polymerase that is bound. Finally, the amount of RNA polymerase bound to ppGpp follows the relationship in Eq. (54). Using population level data for RNA mass fraction, RNA polymerase concentration and ppGpp concentration at different cell doubling times from Bremer and Dennis^30^ allows *K*_*M*_ (as well as overall expression rates from free and ppGpp bound RNA polymerase) to be determined with a least squares fit based on the following relationship:

55 $$RN{A}_{fraction,i}={C}_{RNAP,i}\cdot ((1-{f}_{i})\cdot ex{p}_{free}+{f}_{i}\cdot ex{p}_{ppGpp})$$

56 $${f}_{i}=\frac{{C}_{ppGpp,i}^{2}}{{K}_{M}^{2}+{C}_{ppGpp,i}^{2}}$$

where *RNA*_*fraction*,*i*_ is the mass fraction of the cell that is RNA, *C*_*RNAP*,*i*_ is the cellular concentration of RNA polymerase and *C*_*ppGpp*,*i*_ is the cellular concentration of ppGpp for each growth rate *i*.

ppGpp-dependent fold change data and basal expression data can be used to solve for *exp*_*free*,*j*_ and *exp*_*ppGpp*,*j*_ for each gene, *j*, using data from Sanchez-Vazquez et al.^64^. To ensure consistency with annotated ppGpp regulation, only genes known to be regulated by ppGpp as curated from EcoCyc^29^ and with consistent regulatory direction with the fold change data are regulated by ppGpp in the model. Notable exceptions to this include the addition of negative regulation of all tRNA synthetases that are not annotated on EcoCyc and the rProtein rpmF, which is the only rProtein that is not annotated under ppGpp regulation. The fold change data from Sanchez-Vazuez et al. represents the change from an uninduced (low ppGpp) condition in rich media to a RelA induced (high ppGpp) condition. Assuming the overexpression of RelA leads to ppGpp concentrations that are much greater than *K*_*M*_ so that all RNA polymerases are bound to ppGpp, the fold change can be represented as:

57 $$F{C}_{j}={\log }_{2}\frac{ex{p}_{induced,j}}{ex{p}_{uninduced,j}}={\log }_{2}\frac{ex{p}_{ppGpp,j}}{(1-{f}_{rich})\cdot ex{p}_{free,j}+{f}_{rich}\cdot ex{p}_{ppGpp,j}}$$

where *FC*_*j*_ is the measured fold change for gene *j* and *f*_*rich*_ is the fraction of RNA polymerases bound to ppGpp in rich media as determined by Eq. (56) with the concentration for ppGpp in rich media.

Measured RNA expression in M9+ glucose conditions (basal) provides another relationship between measured data and *exp*_*free*,*j*_ and *exp*_*ppGpp*,*j*_ for each gene:

58 $$ex{p}_{basal,j}=(1-{f}_{basal})\cdot ex{p}_{free,j}+{f}_{basal}\cdot ex{p}_{ppGpp,j}$$

where *exp*_*basal*,*j*_ is the measured RNA expression data for each gene, *j*, and *f*_*basal*_ is the fraction of RNA polymerases bound to ppGpp in basal media as determined by Eq. (56) with the concentration for ppGpp in basal media.

Taken together, Eq. (57) and Eq. (58) can be used to solve for the unknown values *exp*_*free*,*j*_ and *exp*_*ppGpp*,*j*_. This provides expression for nearly all genes but some adjustments are needed. First, some genes do not have fold change data (transcript is too small, difference was not significant, etc) but are annotated as being regulated by ppGpp. For those with positive regulation, the average fold change of all positively regulated genes, *FC*_+_, is used. For those with negative regulation, the fold change calculated from fitting *exp*_*free*_ and *exp*_*ppGpp*_ in Eq. (55), *FC*_−_, is used since this mostly represents the change in rRNA and tRNA expression which is not measured in the fold change data set. Another adjustment is needed for certain genes with high positive fold changes. In these cases, solving the two equations results in a negative value for *exp*_*free*,*j*_. Since negative RNA expression is not possible, these values are truncated at 0.

Expression adjustments to ribosome and RNA polymerase related genes are currently done outside the framework of ppGpp regulation in order to get the appropriate doubling time in given conditions. To match ppGpp regulation expression levels to these expression adjustments, a least squares fit is performed to determine new expression values for the adjusted genes. Based on Eq. (53), a system of equations can be set up for the expression in each of three conditions (rich, basal, anaerobic):

59 $$F\cdot r=e$$

60 $$\left[\begin{array}{cc}1-{f}_{rich}&{f}_{rich}\\ 1-{f}_{basal}&{f}_{basal}\\ 1-{f}_{anaerobic}&{f}_{anaerobic}\end{array}\right]\cdot \left[\begin{array}{c}ex{p}_{free,j}\\ ex{p}_{ppGpp,j}\end{array}\right]=\left[\begin{array}{c}ex{p}_{rich,j}-t{f}_{rich,j}\\ ex{p}_{basal,j}-t{f}_{basal,j}\\ ex{p}_{anaerobic,j}-t{f}_{anaerobic,j}\end{array}\right]$$

where *f*_*rich*_, *f*_*basal*_ and *f*_*anaerobic*_ are the fractions of RNA polymerase bound to ppGpp in different conditions, *exp*_*free*,*j*_ and *exp*_*ppGpp*,*j*_ are the gene specific expression values for free and ppGpp bound RNA polymerases, *exp*_*rich*,*j*_, *exp*_*basal*,*j*_ and *exp*_*anaerobic*,*j*_ are adjusted expression values for genes of interest in each of the conditions and *tf*_*condition*,*j*_ is the contribution to expression that is expected to be controlled by transcription factors as defined below:

61 $$t{f}_{condition,j}=\frac{ex{p}_{condition,j}\cdot {{\Delta }}{r}_{condition,j}}{{p}_{condition,j}}$$

where Δ*r*_*condition*,*j*_ is the change in probability expected from the average transcription factor binding in the condition as described above and *p*_*condition*,*j*_ is the expected synthesis probability in the condition (not considering ppGpp regulation). This calculation assumes that the ratio between expression and synthesis probability will be constant for each gene, which can be used to convert the expected change in synthesis probability from transcription factors to an expected change in expression. Finally, solving with least squares provides the following solution:

62 $$\hat{r}={({F}^{T}F)}^{-1}{F}^{T}e$$

Additionally, we want the probability of synthesis in minimal media with ppGpp and with transcription factor effects to match the synthesis probability in minimal media without ppGpp regulation, which is the condition used to parameterize transcription factor effects. By doing this, we ensure that transcription factors have the correct magnitude of effect under ppGpp regulation. We need to make adjustments to *exp*_*free*,*j*_ and *exp*_*ppGpp*,*j*_ to scale the probabilities from Eq. (50) (*v*_*synth*,*o*_) and Eq. (51) (*v*_*synth*,*ppGpp*_) to be equal:

63 $$scal{e}_{j}=\frac{{v}_{synth,o,j}}{{v}_{synth,ppGpp,j}}$$

adjusting both *exp*_*free*,*j*_ and *exp*_*ppGpp*,*j*_ by *scale*_*j*_ for any genes that are regulated by transcription factors will ensure that *v*_*synth*,*ppGpp*,*j*_ and *v*_*synth*,*o*,*j*_ are equal.

During simulations, *α*_*j*_ in Eq. (50) becomes dependent on ppGpp concentrations as shown with the equations below:

64 $${\alpha }_{j}=\frac{ex{p}_{j}\cdot loss}{{n}_{genes}}$$

where *exp*_*j*_ is defined in Eq. (53). *loss* is the expected loss rate of the given transcript approximated by:

65 $$loss=\frac{\ln (2)}{\tau }+\frac{\ln (2)}{{t}_{1/2}}$$

where *τ* is the expected doubling time from the current concentration of ppGpp (interpolation performed from data from Bremer and Dennis^30^) and *t*_1/2_ is the measured RNA half life. *n*_*genes*_ is the expected gene copy number which is a function of *τ* as determined above and the gene’s position in the genome.

#### Network component analysis for transcriptional regulation

Although transcriptomics data is widely available in many environmental conditions with new data being produced at a rapid pace, representing the data and changes in expression such that it is consistent with the annotated regulatory network and in a manner that can be incorporated in the whole-cell model can be challenging. For this problem, we used network component analysis (NCA) to greatly expand the number of regulator (transcription factors and uncharged tRNAs) to gene regulatory pairs that are included in the model. In order to parameterize the effect of a regulator on a gene in the model, we need to start with an expected fold change that captures the difference between an inactive and an active regulatory condition. NCA uses gene expression data (*E*) and an interaction network topology (*Z*_*o*_) to decompose the expression data into regulatory interaction strengths between each regulator and gene (*A*) and the overall activity signal for a regulator in each condition (*P*):

66 $$\mathop{\min }\limits_{A,P}\parallel E-A\cdot P{\parallel }^{2}$$

67 $$st.A\in {Z}_{o}$$

For the analysis performed here, gene expression data (*E*) comes from the EcoMAC compendium^28^, which provides log2, normalized gene expression for 4189 genes in 2198 conditions. The network topology (*Z*_*o*_) that only allows specified network connections to be non-zero in *A* comes from EcoCyc annotations^29^. There are several algorithms that can be used to solve the NCA problem, but we used an implementation of Robust NCA^26^ with the option of performing Iterative Sub-NCA^27^ for overlapping regulatory units that would otherwise not have a solution.

Preprocessing of the network topology from EcoCyc is performed to better account for biological differences between activation and repression from regulators. Any dual regulators are split into two separate entries in *Z*_*o*_, one for positively regulated genes and one for negatively regulated genes. Any genes with ambiguous regulation from a regulator are added to both the positively and negatively regulated subset. This approach can improve the fit by allowing the same regulator to have different amounts of positive or negative activity in each condition.

Output from the algorithm will provide a unique solution up to a scaling factor provided that certain criteria are met^25^. In order to convert output to the format we can use in the model (fold changes from one condition to another), we need to consider both *A* and *P* since the magnitude in *A*, which reflects the interaction strengths between a regulator and gene, will be dependent on the range of activities in *P*. To calculate the fold change, we determine an average regulator activity for a set of conditions with high activity (*P*_*j*,*high*_) and a set of conditions with low activity (*P*_*j*,*low*_). The sets are defined as the activities that are more than a standard deviation away from the mean of all condition activities for that regulator and include the top or bottom 10 activities even if these do not fall one standard deviation away from the mean. Using a minimum of 10 conditions limits the impact of outliers for some regulators. This leads to a calculation for the fold change (*FC*_*ij*_) expected for regulator *j* on gene *i*:

68 $$F{C}_{ij}={A}_{ij}\cdot ({P}_{j,high}-{P}_{j,low})$$

#### Transcriptional attenuation

Although transcriptional attenuation represents a relatively small number of regulatory interactions in the cell, it plays an important role in amino acid biosynthesis and tRNA charging stability. When levels of charged tRNA are low, more mRNA for synthesis enzymes will be produced leading to higher enzyme concentrations and higher rates of amino acid synthesis, which can lead to an increase in charging. Conversely, when charged tRNA concentrations are high, enzyme expression is attenuated to prevent excess resources going to the enzymes and the production of amino acids. With the application of NCA to parameterize the magnitude of transcriptional regulation along with dynamic tRNA concentrations in the cell, we can now simulate transcriptional attenuation rates. Attenuation is modeled based on a Poissonian process to determine whether or not transcription proceeds past the attenuation site^65^:

69 $${P}_{stop,i}=1-{{\rm{e}}}^{-{\sum }_{j}\frac{tRN{A}_{j}}{{K}_{i,j}}}$$

where *P*_*stop*_ is the probability that transcriptional attenuation leads to the termination of transcription, *tRNA* is the concentration of charged tRNA and *K* is a parameter calculated from expression data to represent the strength of attenuation for a tRNA species *j* on transcription of gene *i*. tRNA species for each amino acid are treated as the same so their concentrations are summed together and they have the same *K* value.

*K* values are calculated before simulations using expected tRNA concentrations in rich media and fold changes calculated by applying the NCA method to the transcriptional attenuation regulatory interactions specified on EcoCyc^29^. The fold change is assumed to represent the change in transcriptional activity from a condition with no charged tRNA to a condition with the highest expected charged tRNA:

70 $$F{C}_{i}=\frac{1-{P}_{stop,i}(tRN{A}_{rich})}{1-{P}_{stop,i}(tRNA=0)}$$

where *FC* is the expected fold change. Since *P*_*stop*,*i*_ = 0 when there are no charged tRNA, we can simplify the equation and solve for *K* directly:

71 $$F{C}_{i,j}=1-{P}_{stop,i}(tRN{A}_{rich})$$

72 $$F{C}_{i,j}={{\rm{e}}}^{-\frac{tRN{A}_{j,rich}}{{K}_{i,j}}}$$

73 $${K}_{i,j}=\frac{tRN{A}_{j,rich}}{\ln F{C}_{i}}$$

With transcriptional attenuation, the number of transcripts will be lower than previously calculated. Basal synthesis probability parameters are calculated based on measured expression without accounting for transcriptional attenuation so we must adjust these higher. Calculating the adjustment parameter is done prior to simulations and a new *α* for Eq. (50) or Eq. (51) is defined as

74 $${\alpha }_{j,adjusted}={\alpha }_{j}+adjust$$

where *adjust* is calculated such that the basal probability with the adjustment and transcriptional attenuation is the same as the original basal probability:

75 $$\left(1-{P}_{stop}\right)\cdot {\alpha }_{j,adjusted}={\alpha }_{j}$$

rearranging, we can solve for *adjust*:

76 $$adjust={\alpha }_{j}\cdot \left(\frac{1}{1-{P}_{stop}}-1\right)$$

During the simulation, the initiation probability of genes controlled by transcriptional attenuation will use the adjusted basal probability (*α*_*j*,*adjusted*_). Additionally, at each time step of the simulation, we calculate the stop probability of each actively transcribing RNAP if it is transcribing a gene under transcriptional attenuation control by using the charged tRNA concentration in the cell. A random number is drawn from a uniform distribution and if it is less than the calculated probability, then we remove the RNAP from DNA and transcription of that gene stops.

#### Other modeling updates

The changes listed below indicate updated functionality compared to what existed in the previous version of the model. This does not include new features like amino acid biosynthesis kinetics, ppGpp kinetics, transcriptional attenuation or tRNA charging, which were discussed in the previous section, but does include how those new features have impacted previous implementations with new functionality.

- Metabolism:
  - Metabolite concentrations now come from an average from literature with new datasets added^22,35,66,67^, including concentrations for 38 new metabolites.
  - ppGpp concentration is now available and dynamic based on kinetic equations (see more above).
  - Amino acid concentrations are dynamic based on supply and demand, which are determined by the simulation state (see more above).
  - Biomass-related metabolite concentrations are now determined based on the RNA to protein ratio of the simulation instead of the expected doubling time of the condition.
  - Dynamic s-adenosylmethionine (SAM) concentration based on methionine concentration.
  - cAMP concentration is a step function for known conditions based on measured relative changes^22^.
- Regulation:
  - Additional transcription factors have been added (Crp, LrhA).
  - Additional transcription factor to gene interactions have been added (see more above).
  - Variable amino acid and SAM concentrations leads to variable transcription factor activity for ArgP, ArgR, Lrp, MetJ, PutA, TrpR, and TyrR.
  - Transcription factor binding occurs before other processes in a timestep to more accurately calculate binding.
  - Active forms, effector binding and gene regulation direction for some transcription factors (ArgR, Lrp, TyrR) have been updated to more accurately capture the biological effects in the model.
- Values previously modeled as constants unique to each environment based on experimentally measured values and the expected simulation doubling time, which resulted in step functions between conditions and the inability to simulate outside of parameterized/experimentally characterized conditions:
  - RNAP active fraction determined from ppGpp binding RNAP.
  - Fraction of RNA types synthesized (mRNA, rRNA, tRNA) is determined from ppGpp binding RNAP and transcription factors.
  - Fraction of mRNA synthesized that is rProtein and RNAP is determined from ppGpp binding RNAP and transcription factors.
  - A variable transcription elongation rate is used for stable RNA elongation and mRNA elongation.
  - Amino acid supply rates to translation are determined by amino acid, biosynthesis enzyme, transporter, tRNA synthetase and tRNA concentrations (see more above).
  - The ribosome elongation rate is determined by ribosome, tRNA and ppGpp concentrations (see more above).
- New environments:
  - New carbon sources: acetate and succinate.
  - Single amino acid addition to minimal media or removal from rich media.
  - Other arbitrary amino acid combinations.

### Simulated experiments

#### Simulation descriptions

All simulations were performed by initializing a given number of cells with a different random seed. Simulations continued for a defined number of cell cycles with one of the two daughter cells providing the initial conditions for a new simulated cell cycle after division. Although initialization of simulations produces a cell state that is representative of cell states achieved during simulations, under perturbed conditions this might not be the case. To account for this, the first few generations are excluded from some analysis while the simulation state normalizes as noted below. Due to compute and storage limitations, simulation sets with many variants are often run with fewer generations or initial seeds than simulation sets with fewer variants (eg. Simulation set 16 vs. Simulation set 17). Commands for generating workflows for each set of simulations are are included on GitHub at https://github.com/CovertLab/WholeCellEcoliRelease/blob/v2.0/runscripts/growth-paper/sims.sh. After queueing the workflow with the python runscripts/fireworks/fw_queue.py command, workflows were run with qlaunch -r rapidfire --nlaunches infinite --sleep 5. More details about setting up the computing environment and running simulations is given on the GitHub page: https://github.com/CovertLab/WholeCellEcoliRelease.

#### Media shifts

Simulations were performed with 32 starting seeds grown for 28 generations. Simulations began in rich media (M9 glucose + all amino acids). At 5 hr, media was shifted to minimal media (M9 glucose). At 10 hr, amino acids were added back into the media. Four different combinations of simulation options were used:

**Simulation set 1** All regulation

Simulations include all newly added regulation.

**Simulation set 2** No mechanistic amino acid supply

Simulations do not include mechanistic amino acid supply or transport so that the amount of amino acids supplied to tRNA charging is determined in a phenomenological manner that is independent of enzyme and transporter expression.

**Simulation set 3** No ppGpp regulation

Simulations do not include ppGpp regulation of transcript expression, RNAP properties or GTPase activity (ribosome elongation rate).

**Simulation set 4** No new regulation

Simulations do not include any newly added regulation or kinetics (no ppGpp regulation, no mechanistic amino acid supply or transport, no transcriptional attenuation, and no tRNA charging). This is mainly representative of simulations from the first version of the *E. coli* whole-cell model described in Macklin et al.^16^ with some minor added features and bug fixes. Growth related properties such as RNAP active fraction and ribosome elongation rate follow step functions during environmental shifts based on the expected value from experimental characterization in each condition.

#### Amino acid combinations in media

Simulations were performed with 24 starting seeds grown for 24 generations in glucose minimal media supplemented with different combinations of amino acids - without amino acids, with 6 amino acids (Arg, His, Met, Pro, Thr, Trp), with 12 amino acids (previous 6 plus Ala, Asn, Asp, Leu, Ser, Tyr), and with all amino acids. Simulations with 6 or 12 amino acids started with all amino acids in the media to initialize the simulations with a known environment and after 10 min, the other amino acids were removed. Two combinations of options were used:

**Simulation set 5** All regulation

Simulations include all newly added regulation.

**Simulation set 6** No regulation

Simulations do not include any newly added regulation or kinetics (no ppGpp regulation, no mechanistic amino acid supply or transport, no transcriptional attenuation, and no tRNA charging). This is mainly representative of simulations from the first version of the *E. coli* whole-cell model described in Macklin et al.^16^ with some minor added features and bug fixes. Growth-related properties such as RNAP active fraction and ribosome elongation rate follow step functions during environmental shifts based on the expected value from experimental characterization in each condition.

#### Single amino acid media

Simulations were performed with 4 starting seeds grown for 16 generations. Simulations began in rich or minimal media and an amino acid was removed or added, respectively, after 10 min of growth.

**Simulation set 7** Add one amino acid to M9 glucose

**Simulation set 8** Remove one amino acid from M9 glucose + all amino acids

#### Media conditions

Simulations were performed with 16 starting seeds grown for 8 generations in M9 glucose grown aerobically, M9 glucose grown anaerobically, M9 glucose with all amino acids, M9 acetate, or M9 succinate. Two combinations of options were used:

**Simulation set 9** All regulation

Simulations include all newly added regulation.

**Simulation set 10** No regulation

Simulations do not include any newly added regulation or kinetics (no ppGpp regulation, no mechanistic amino acid supply or transport, no transcriptional attenuation, and no tRNA charging). This is mainly representative of simulations from the first version of the *E. coli* whole-cell model described in Macklin et al.^16^ with some minor added features and bug fixes. Growth-related properties such as RNAP active fraction and ribosome elongation rate follow step functions during environmental shifts based on the expected value from experimental characterization in each condition.

#### ppGpp sensitivity analysis

Simulations were performed with 8 starting seeds grown for 8 generations. ppGpp concentrations were fixed at 10-100 *μ*M in 10 *μ*M increments. Growth was in M9 glucose media or M9 glucose with all amino acids.

**Simulation set 11** ppGpp sensitivity analysis

#### Expression limitations with fixed ppGpp concentrations

Simulations were performed with 4 starting seeds grown for 8 generations on M9 glucose media. Expression for metabolic enzymes or ribosomes was adjusted to determine how expression limited growth at perturbed ppGpp concentrations. ppGpp concentrations started at 50 *μ*M (the expected concentration for minimal media) and had a slow ramp to a perturbed concentration at a rate of 0.01 *μ*M/s for improved stability.

**Simulation set 12** Limitations at low ppGpp

The final concentration of ppGpp was set at 20 *μ*M. Simulations were either a control with no expression changes, had adjustments to expression for amino acid biosynthesis enzymes between 0.5x and 2x, or had adjustments to ribosomal genes (rProtein and rRNA) between 0.5x and 2x.

**Simulation set 13** Limitations at high ppGpp

The final concentration of ppGpp was set at 90 *μ*M. Simulations were either a control with no expression changes, had adjustments to expression for amino acid biosynthesis enzymes between 0.5x and 2x, or had adjustments to ribosomal genes (rProtein and rRNA) between 0.5x and 2x.

**Simulation set 14** Limitations at high ppGpp with variable ribosome expression

The final concentration of ppGpp was set at 90 *μ*M. Simulations were either a control with no expression changes, had adjustments to expression for rRNA at 1.1x, 1.25x or 1.5x, had adjustments to expression for rProtein at 1.1x, 1.25x or 1.5x, had adjustments to expression for both rRNA and rProtein at 1.1x, 1.25x or 1.5x, had adjustments to expression for rRNA at 1.2x, 1.5x or 2x and adjustments to expression for rProtein at 1.1x, 1.25x, or 1.5x, respectively, or had adjustments to expression for rRNA at 1.5x, 2.25x or 3.5x and adjustments to expression for rProtein at 1.1x, 1.25x, or 1.5x, respectively.

**Simulation set 15** Limitations at high ppGpp with variable ribosome expression and no GTPase inhibition

The final concentration of ppGpp was set at 90 *μ*M. Simulations were either a control with no expression changes, had adjustments to expression for rRNA at 1.1x, 1.25x or 1.5x, had adjustments to expression for rProtein at 1.1x, 1.25x or 1.5x, had adjustments to expression for both rRNA and rProtein at 1.1x, 1.25x or 1.5x, had adjustments to expression for rRNA at 1.2x, 1.5x or 2x and adjustments to expression for rProtein at 1.1x, 1.25x, or 1.5x, respectively, or had adjustments to expression for rRNA at 1.5x, 2.25x or 3.5x and adjustments to expression for rProtein at 1.1x, 1.25x, or 1.5x, respectively. The inhibitory interaction between ppGpp and translational GTPases was also removed so that the ribosome elongation rate no longer depended on the concentration of ppGpp.

#### Removing allosteric amino acid inhibition

Simulations were performed in M9 glucose media. Simulations included wildtype cells as well as mutants of ArgA, TrpE, HisG, LeuA, ThrA, IlvA, and ProB with varying levels of amino acid end product allosteric inhibition. Due to computational limitations, two sets of sims were run with a smaller number of cells but more values of inhibition removal or a larger number of cells but only complete inhibition removal as described below:

**Simulation set 16** Partial inhibition removal

Simulations were performed with 4 starting seeds grown for 8 generations. Mutants had multiple levels of inhibition removal determined by increasing the original *K*_*I* parameter by 2x, 5x, 10x, 100x, or *∞*x (complete removal).

**Simulation set 17** Larger set of sims with full inhibition removal

Simulations were performed with 16 starting seeds grown for 16 generations. Mutants had complete inhibition removal.

### Simulation analysis methods

#### Figure 2

*Panels a and b*: Simulation data comes from Simulation set 4 for a and Simulation set 1 for b. Time series data is binned into 5 s bins and averaged to downsample the original data. The mean value for each value is calculated across all simulation seeds for each bin and shown as the solid trace. The standard deviation is calculated across all simulation seeds for each bin and added to or subtracted from the mean to produce the shaded region. All values come directly from simulation outputs at each time step except for a few noted below:

- LEU conc: the model produces counts of leucine and a conversion factor to convert from counts to molar concentration (based on Avogadro’s number and the current cell volume). These values are multiplied together to produce the concentration.
- Fraction charged LEU tRNA: the model provides counts of each leucine tRNA species that is charged and uncharged. The counts of each species that are charged is summed together at each time step to produce a total count of charged tRNA. The counts of each species that are uncharged are also summed together. The counts for charged and uncharged are then added together to produce a total tRNA count specific to leucine. The fraction charged is then calculated as the charged counts divided by total counts.
- ppGpp conc: the model produces counts of ppGpp and a conversion factor to convert from counts to molar concentration (based on Avogadro’s number and the current cell volume). These values are multiplied together to produce the concentration.

*Panels c and d*: Simulation data comes from Simulation set 6 for c and Simulation set 5 for d. The model provides growth rate at each time step and this value is averaged over every 100 time steps before producing the histograms. Data from the first 6 simulation generations is dropped to provide sufficient time for the simulation to reach a new steady state when shifting from rich media to only a subset of the amino acids in the media.

*Panel e*: Literature data (dashed line) comes a linear fit to data in Bremer et al.^30^. Simulation data representative of the simulation output from the previous version of the model in three possible media conditions comes from Simulation set 10, data for the new model in parameterized conditions comes from Simulation set 9 (excluding the first 6 generations) and Simulation set 5, and data for the new model in unparameterized conditions comes from Simulation set 7 (excluding the first 2 generations) and Simulation set 8 (excluding the first 4 generations). Growth rate comes directly from simulation output. RNA/protein mass ratio is determined by the RNA mass divided by protein mass at each time step. A moving average is applied to each cell trajectory data series (multiple generations from a single starting seed) with a window of 200 time points. The mean and standard deviation is then calculated for the plot with error bars representing a standard deviation. Pearson’s r between the growth rate and RNA/protein mass ratio is calculated for the groups of points indicated on the plot and squared to produce the *R*^2^ shown on the plot.

*Panel f*: Literature data comes from the maximum rate observed in Supplemental Data 1 from Zampieri et al.^31^. The model uptake flux is calculated prior to sims as the expected uptake flux to meet demands of translation, which is used to calculate transport parameters used in the sim. Pearson’s r between the model and literature data is calculated and squared to produce the *R*^2^ shown on the plot.

#### Figure 3

*Panel a*: Literature data comes from Fig. 2d (increasing ppGpp) and 3H (decreasing ppGpp) from Zhu et al.^32^. Additionally, a linear fit is shown for data in Bremer et al.^30^. Simulation data for perturbed ppGpp conditions comes from Simulation set 11 (excluding the first 2 generations to allow for normalization to the adjusted ppGpp concentration). Growth rate comes directly from simulation output. RNA/protein mass ratio is determined by the RNA mass divided by protein mass at each time step. A moving average is applied to each cell trajectory data series (multiple generations from a single starting seed) with a window of 200 time points before the mean across all values is taken.

*Panels b, c, d, e, f, h and i*: Simulation data comes from Simulation set 11 (excluding the first 2 generations to allow for normalization to the adjusted ppGpp concentration). Growth rate and elongation rate (per ribosome) come directly from simulation output. Other values are calculated based on simulation outputs:

- Normalized capacity (ribosomes): capacity in each condition as defined below normalized by the capacity at 50 *μ*M.
  77 $$capacity={k}_{cat,max}\cdot ({N}_{rib,active}+{N}_{rib,inactive})$$
  where *k*_*cat*,*max*_ is the maximum ribosome elongation rate, *N*_*rib*,*active*_ is the number of active ribosomes, and *N*_*rib*,*inactive*_ is the number of inactive ribosomes.
- Normalized capacity (amino acid enzymes): capacity in each condition as defined below normalized by the capacity at 50 *μ*M.
  78 $$capacity=\mathop{\sum}\limits_{AA}{k}_{cat,fwd,AA}\cdot {N}_{enz,AA}$$
  where *k*_*cat*,*fwd*,*AA*_ is the forward reaction rate and *N*_*enz*,*AA*_ is the number of enzymes to catalyze the reaction for each amino acid, *AA*.
- Output (ribosomes): the model produces counts of amino acids elongated by the ribosome and a conversion factor to convert from counts to molar concentration (based on Avogadro’s number and the current cell volume). These values are multiplied together to produce the concentration and normalized by the time step to produce a rate.
- Output (amino acid enzymes): the model produces counts of amino acids produced and a conversion factor to convert from counts to molar concentration (based on Avogadro’s number and the current cell volume). These values are multiplied together to produce the concentration and normalized by the time step to produce a rate.
- rRNA excess: the model produces counts of rRNA, ribosome subunits and ribosomes. After converting these counts to a mass basis with molecular weights, we divide the mass of rRNA not in the ribosome by the ribosome mass.
- Average GTPase inhibition: the model produces counts of ppGpp and a conversion factor to convert from counts to molar concentration (based on Avogadro’s number and the current cell volume). These values are multiplied together to produce the concentration ([*ppGpp*]) for the calculation below with parameters *K*_*I*_ and *H*:
  79 $$inhibition=1-\frac{1}{1+{\left(\frac{[ppGpp]}{{K}_{I}}\right)}^{H}}$$
- Total AA conc: the model produces counts of amino acids in the cell and a conversion factor to convert from counts to molar concentration (based on Avogadro’s number and the current cell volume). These values are multiplied together to produce the concentration summed over all amino acids.
- Average allosteric inhibition: the model produces counts of amino acids in the cell and a conversion factor to convert from counts to molar concentration (based on Avogadro’s number and the current cell volume). These values are multiplied together to produce the concentration (*AA*_*i*_) used with the inhibition constant for each amino acid (*K*_*I*,*i*_) to calculate inhibition for each amino acid pathway as shown below. The mean of this value for all amino acids is reported on the plot.
  80 $$inhibitio{n}_{i}=1-\frac{1}{1+\frac{[A{A}_{i}]}{{K}_{I,i}}}$$

*Panel g*: Simulation data comes from Simulation set 11 (Con) and Simulation set 12 (Enz, Rib) (excluding the first 2 generations to allow for normalization to the adjusted ppGpp concentration). The model produces growth rate, which is averaged over all time steps.

*Panel j*: Simulation data comes from Simulation set 11 (Con), Simulation set 13 (Enz), Simulation set 14 (Rib), Simulation set 15 (GTP and Rib/GTP) (excluding the first 2 generations to allow for normalization to the adjusted ppGpp concentration). The model produces growth rate, which is averaged over all time steps.

#### Figure 4

*Panel b*: Experimental data comes from Fig. 2b and supplement from Sander et al.^35^. Simulation data comes from Simulation set 17. The model produces counts of amino acids in the cell and a conversion factor to convert from counts to molar concentration (based on Avogadro’s number and the current cell volume). These values are multiplied together to produce the concentration.

*Panel c*: Simulation data comes from Simulation set 17. The model produces counts of amino acids in the cell and a conversion factor to convert from counts to molar concentration (based on Avogadro’s number and the current cell volume). These values are multiplied together to produce the concentration. Time series data are trimmed to 5 h. After calculating the concentration in each cell, the mean is taken across all cells that were simulated.

*Panel d*: Simulation data comes from Simulation set 17. The model produces counts of amino acids in the cell and a conversion factor to convert from counts to molar concentration (based on Avogadro’s number and the current cell volume). These values are multiplied together to produce the concentration. Wildtype represents the amino acid concentrations for all the amino acids listed from the wildtype simulations without enzyme modification. The mutant for each amino acid comes from a different set of sims representing the modified enzyme corresponding to the amino acid listed (Arg: ArgA mutant, Trp: TrpE mutant, His: HisG mutant, Leu: LeuA mutant, Thr: ThrA mutant, Ile: IlvA mutant, Pro: ProB mutant).

Concavity changes are calculated from each individual cell trajectory. First concentrations are binned and averaged over every 5 timesteps. Next, a moving average is applied to the downsampled concentrations with a window of 60 timesteps (5 min). Then, we calculate a cubic spline interpolation and take the second derivative to represent the concavity of the concentration time series. Finally, we take a moving average of the second derivative and calculate number of times this value changes sign. This number of changes is divided by the simulation length in hr to get the rate of change. This value is averaged over all cell trajectories and shown in the figure.

*Panel e*: Experimental data comes from Fig. 2b and supplement from Sander et al.^35^ and is the concentration of the amino acid in the corresponding mutant (Arg: ArgA mutant, Trp: TrpE mutant, His: HisG mutant, Leu: LeuA mutant, Thr: ThrA mutant, Ile: IlvA mutant, Pro: ProB mutant) divided by the amino acid concentration in the wildtype. Simulation data comes from Simulation set 16. The model produces counts of amino acids in the cell and a conversion factor to convert from counts to molar concentration (based on Avogadro’s number and the current cell volume). These values are multiplied together to produce the concentration at each time step. The mean concentration across all time steps within wildtype simulations and for simulations with each level of inhibition in the mutants (adjusted *K*_*I*_ is 2x, 5x, 10x, 100x, or *∞*x the original *K*_*I*_). A cubic spline interpolation in log space for the plotted x and y points is calculated for each concentration. This interpolation is plotted as the solid line and used to calculated the predicted Original *K*_*I*_ / adjusted *K*_*I*_ based on the experimentally observed fold change.

*Panel f*: Wildtype *K*_*I*_ parameters come from literature. Mutant *K*_*I*_ values are calculated form the wildtype *K*_*I*_ values by dividing by the predicted original *K*_*I*_ / adjusted *K*_*I*_ calculated in (e).

#### Figure 5

*Panels a, b and c*: Literature data (dashed line) comes a linear fit to data in Bremer et al.^30^. Simulation data for minimal and rich media references (single points) comes from Simulation set 9 (excluding the first 6 generations). Simulation data for the trajectories comes from Simulation set 1 for a, Simulation set 2 for b, and Simulation set 3 for c. Growth rate comes directly from simulation output. RNA/protein mass ratio is determined by the RNA mass divided by protein mass at each time step. A moving average is applied to each cell trajectory data series (multiple generations from a single starting seed) with a window of 200 time points. The mean and standard deviation for the reference points is then calculated, with error bars representing a standard deviation.

*Panels d, e and f*: Simulation data comes from Simulation set 1 for d, Simulation set 2 for e, and Simulation set 3 for f. The model provides total RNA and protein mass at each time step. The change in mass in each time step is used to calculate the growth rate as below. The data plotted is downsampled from the raw data by binning growth rates every 10 time steps and taking the average from the 10 time steps.

81 $$growth=\frac{mas{s}_{t+1}-mas{s}_{t}}{mas{s}_{t}\cdot {{\Delta }}t}$$

*Panel g, h and I*: Simulation data comes from Simulation set 1 for All regulation and Simulation set 3 for No ppGpp.

- RNAP output: the model produces counts of nucleotides elongated by RNAPs and a conversion factor to convert from counts to molar concentration (based on Avogadro’s number and the current cell volume). These values are multiplied together to produce the concentration and normalized by the time step to produce a rate.
- RNA degradation rate: the model produces a mass of total RNA degraded in a time step and the total RNA mass. These values are used with the time step size to calculate the rate as below:
  82 $$rate=\frac{mas{s}_{deg}}{mas{s}_{rna}\cdot {{\Delta }}t}$$
- mRNA:rRNA ratio: the model produces a total mass of mRNA and rRNA at each time step. These values are divided to produce the ratio between mRNA and rRNA.

## Supplementary information

Supplemental Material

## Data Availability

See the GitHub repository (https://github.com/CovertLab/WholeCellEcoliRelease) for code and scripts used to generate simulation data and figures.

## Code availability

All code is available at https://github.com/CovertLab/WholeCellEcoliRelease.
