# Supplementary material for: An expanded whole-cell model of E. coli links cellular physiology with mechanisms of growth rate control
Source: NPJ Syst Biol Appl. 2022 Aug 19;8:30. doi: 10.1038/s41540-022-00242-9 (PMC9391491; doi:10.1038/s41540-022-00242-9)
Supplement: Supplementary file 1 — Supplemental Material [file 41540_2022_242_MOESM1_ESM.pdf]

Supplemental materials for an expanded whole-cell model of *E. coli* links cellular physiology with mechanisms of growth rate control

Travis A. Ahn-Horst[1], Luis Santiago Mille[1], Gwanggyu Sun[1], Jerry H. Morrison[1], Markus W. Covert[1*]

npj Systems Biology and Applications

1: Department of Bioengineering, Stanford University, Stanford, CA 94305

*: corresponding author, mcovert@stanford.edu

This PDF file includes:

Supplementary Figures 1 - 2

Supplementary Tables 1 - 4

References

1 Supplementary figures

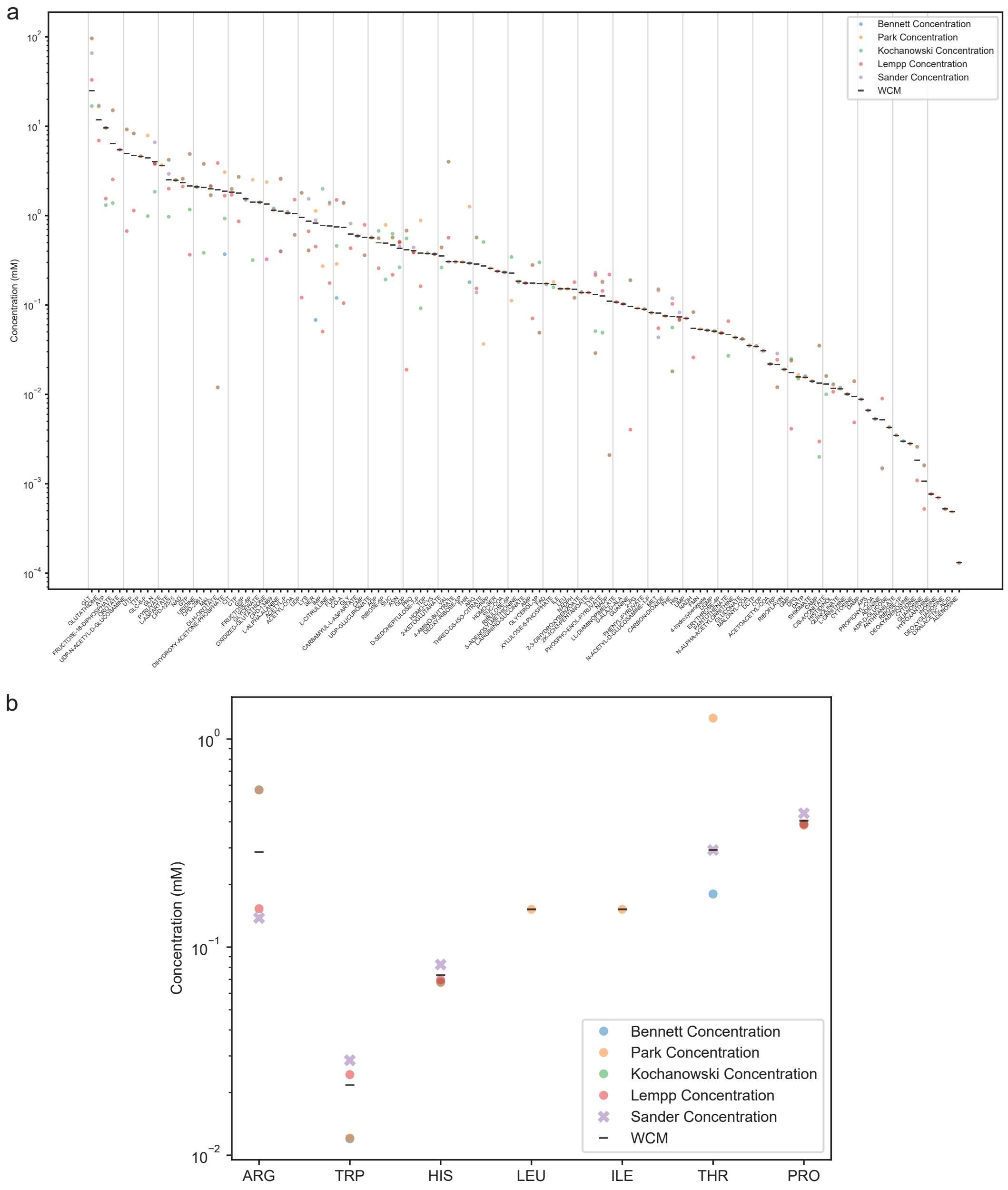

Supplementary Figure 1: a. Concentrations of metabolites measured in literature (circle or x) (1; 2; 3; 4; 5) and the average value that is used as the expected concentration in the whole cell model (bar). b. A selected subset of (a) to highlight the amino acids in the dysregulated pathways explored by introducing mutant enzymes that lack allosteric feedback in Sander *et al.* (5) and our simulations

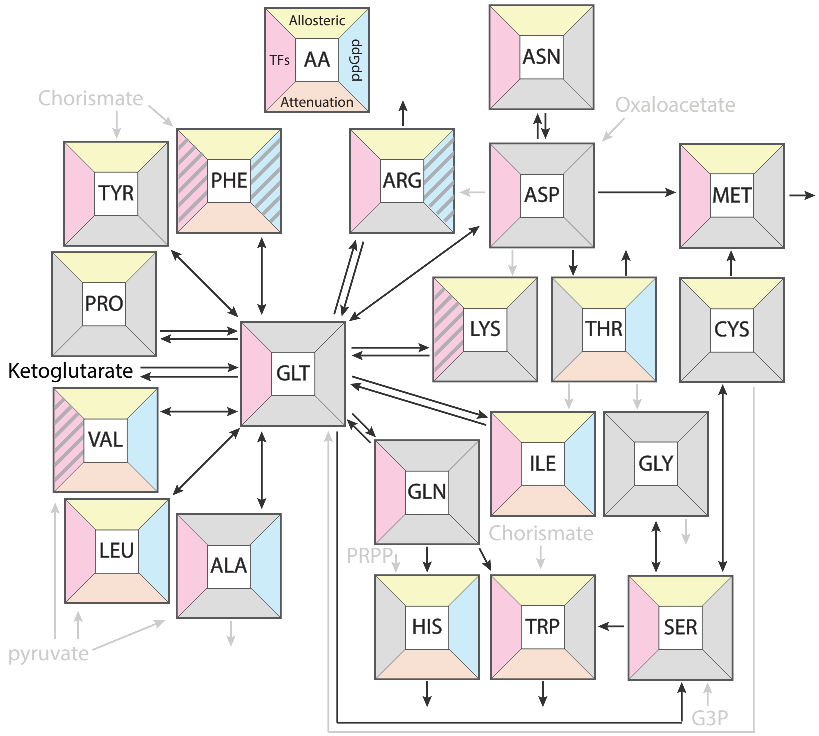

Supplementary Figure 2: Representation of the amino acid network implemented. Highlighted panels indicate the regulation that the reaction experiences from other amino acids (allosteric inhibition, transcription factors or transcriptional attenuation) or ppGpp. Arrows indicate directionality of reactions. Gray arrows indicate annotated reactions that are not yet included in this kinetic reaction network.

2 Supplementary tables

	Previous model			This model		
	Regulators	Genes	Interactions	Regulators	Genes	Interactions
Transcription factors	22	316	384	24	1101	1512
ppGpp	-	-	-	1	394	394
Transcriptional attenuation	-	-	-	7	31	43
Total regulation	22	316	384	32	1368	1949

Supplementary Table 1: A comparison of the transcriptional regulation included in the previous version of the model and this work. Interactions represent distinct regulatory control between a regulator (transcription factor, ppGpp, or tRNA species) and a specific gene. Note that multiple genes are regulated by more than one type of regulation so the number of genes under Total Regulation is not a direct sum of the column.

Amino acid	$k_{cat,fwd}$ (1/s)	$k_{cat,rev}$ (1/s)	K_I (μM)	Upstream K_M (mM)	Reverse K_M (mM)	Degradation K_M (mM)
ALA	38.3	312		GLT: 24.9	4.9	
ARG	850	355	150	GLT: 2.65	2.87	
ASN	79.7	44	120	ASP: 2.53	4	
ASP	320	1600		GLT: 24.9	25.3	
CYS	22800	85.4	1	SER: 1.17	0.217	
GLT	5540	5590				2.3
GLN	41.5	0.0104		GLT: 3.3	0.25	
GLY	444	526		SER: 0.14	0.25	
HIS	22.1	131	73.3	GLN: 0.24		2.57
ILE	3950	3.68	60	GLT: 24.9	0.52	
LEU	80	3.53	280	GLT: 24.9	0.58	
LYS	85	577	871	GLT: 1.21	8.71	
MET	72.3	12.2	100	ASP: 0.97, CYS: 0.05		0.11
PHE	38.7	4.2	100	GLT: 24.9	0.333	
PRO	619	17.3	150	GLT: 24.9	2.7	
SER	1540	31300	370	GLT: 24.9	8.22	
THR	94	42.6	167	ASP: 0.97		6.5
TRP	78.7	3.9	170	GLN: 4.02, SER: 0.822		0.12
TYR	459	0.00473	100	GLT: 24.9	0.625	
VAL	236	0.066	100	GLT: 24.9	3.13	

Supplementary Table 2: Kinetic parameters for the amino acid biosynthesis network.

Amino acid	k_{cat} import (1/s)	k_{cat} export (1/s)	Import K_I (mM)	Export K_M (mM)
ALA	397	213	2.23	40
ARG	15400	310	6.58	82.8
ASN	3750	19.5	0.863	125
ASP	1390	299	5.07	732
CYS	0	0	0.0434	6.27
GLT	451	22.4	49.8	7190
GLN	55	2.64	8.04	1160
GLY	1440	38.7	1.25	180
HIS	6700	10.6	0.147	21.2
ILE	435	13.4	0.304	70.5
LEU	511	43.7	0.341	40.5
LYS	153	90.6	12.7	252
MET	12600	7.23	0.162	40
PHE	174	41.4	0.148	7
PRO	291	14.7	0.81	117
SER	4080	512	1.64	238
THR	4130	5.61	0.586	92.5
TRP	1270	55	0.388	6.27
TYR	1400	129	1.88	37.9
VAL	723	14.1	0.61	108

Supplementary Table 3: Kinetic parameters for amino acid transport.

Amino acid	Wildtype	argA	trpE	hisG	leuA	thrA	ilvA	proB
ARG	0.32 (0.017)	5.4 (1.3)	0.27 (0.043)	0.35 (0.04)	0.29 (0.043)	0.31 (0.034)	0.31 (0.058)	0.27 (0.021)
TRP	0.032 (0.027)	0.068 (0.088)	0.26 (0.12)	0.047 (0.046)	0.066 (0.034)	0.026 (0.032)	0.016 (0.015)	0.022 (0.013)
HIS	0.037 (0.0052)	0.035 (0.011)	0.035 (0.0089)	5.6 (4.2)	0.041 (0.017)	0.035 (0.0081)	0.033 (0.0082)	0.038 (0.0085)
ILE	0.13 (0.024)	0.21 (0.11)	0.15 (0.038)	0.12 (0.019)	0.078 (0.019)	0.1 (0.034)	160 (51)	0.12 (0.04)
LEU	0.1 (0.012)	0.1 (0.024)	0.11 (0.021)	0.12 (0.024)	3.1 (1.1)	0.098 (0.023)	0.095 (0.0094)	0.098 (0.024)
THR	0.24 (0.062)	0.24 (0.092)	0.27 (0.041)	0.27 (0.085)	0.22 (0.042)	95 (26)	0.27 (0.062)	0.3 (0.082)
PRO	0.46 (0.028)	0.44 (0.086)	0.36 (0.022)	0.43 (0.11)	0.37 (0.057)	0.4 (0.1)	0.42 (0.096)	100 (26)

Supplementary Table 4: Mean and standard deviation in parentheses for amino acid concentrations in wildtype and mutant simulations. Values are in units of mM.

References

[1] Bryson D Bennett, Elizabeth H Kimball, Melissa Gao, Robin Osterhout, Stephen J Van Dien, and Joshua D Rabinowitz. Absolute metabolite concentrations and implied enzyme active site occupancy in escherichia coli. *Nature chemical biology*, 5(8):593–599, 2009.

[2] Junyoung O Park, Sara A Rubin, Yi-Fan Xu, Daniel Amador-Noguez, Jing Fan, Tomer Shlomi, and Joshua D Rabinowitz. Metabolite concentrations, fluxes and free energies imply efficient enzyme usage. *Nature chemical biology*, 12(7):482–489, 2016.

[3] Karl Kochanowski, Luca Gerosa, Simon F Brunner, Dimitris Christodoulou, Yaroslav V Nikolaev, and Uwe Sauer. Few regulatory metabolites coordinate expression of central metabolic genes in escherichia coli. *Molecular Systems Biology*, 13(1):903, 2017.

[4] Martin Lempp, Niklas Farke, Michelle Kuntz, Sven Andreas Freibert, Roland Lill, and Hannes Link. Systematic identification of metabolites controlling gene expression in e. coli. *Nature communications*, 10(1):1–9, 2019.

[5] Timur Sander, Niklas Farke, Christoph Diehl, Michelle Kuntz, Timo Glatter, and Hannes Link. Allosteric feedback inhibition enables robust amino acid biosynthesis in e. coli by enforcing enzyme overabundance. *Cell systems*, 8(1):66–75, 2019.
